# Fibroblast growth factor-2 bound to specific dermal fibroblast-derived extracellular vesicles is protected from degradation

**DOI:** 10.1038/s41598-022-26217-8

**Published:** 2022-12-22

**Authors:** Isabelle Petit, Ayelet Levy, Soline Estrach, Chloé C. Féral, Andrea Gonçalves Trentin, Florent Dingli, Damarys Loew, Jieqiong Qu, Huiqing Zhou, Clotilde Théry, Céline Prunier, Daniel Aberdam, Olivier Ferrigno

**Affiliations:** 1 Sorbonne Université, Inserm U938, Centre de Recherche Saint-Antoine, 75012 Paris, France; 2 INSERM U976, Hôpital St-Louis, 75010 Paris, France; 3 Université Côte d’Azur, INSERM, CNRS, IRCAN, Faculté de Médecine, 06107 Nice, France; 4 Federal University of Santa Catarina, Florianópolis, Brazil; 5 Institut Curie, PSL Research University, Centre de Recherche, Laboratoire de Spectrométrie de Masse Protéomique, 75005 Paris, France; 6 Department of Molecular Developmental Biology, Faculty of Science, Radboud Institute for Molecular Life Sciences, Radboud University, 6525GA Nijmegen, The Netherlands; 7 Department of Human Genetics, Radboud Institute for Molecular Life Sciences, Radboud University Medical Center, 6525GA Nijmegen, The Netherlands; 8 INSERM U932, Institut Curie, PSL Research University, 75005 Paris, France; 9 Université de Paris, INSERM U1138, Centre des Cordeliers, 75006 Paris, France

**Keywords:** Cell biology, Extracellular signalling molecules, Growth factor signalling, Cytokines

## Abstract

Fibroblast growth factor-2 (FGF2) has multiple roles in cutaneous wound healing but its natural low stability prevents the development of its use in skin repair therapies. Here we show that FGF2 binds the outer surface of dermal fibroblast (DF)-derived extracellular vesicles (EVs) and this association protects FGF2 from fast degradation. EVs isolated from DF cultured in the presence of FGF2 harbor FGF2 on their surface and FGF2 can bind purified EVs in absence of cells. Remarkably, FGF2 binding to EVs is restricted to a specific subpopulation of EVs, which do not express CD63 and CD81 markers. Treatment of DF with FGF2-EVs activated ERK and STAT signaling pathways and increased cell proliferation and migration. Local injection of FGF2-EVs improved wound healing in mice. We further demonstrated that binding to EVs protects FGF2 from both thermal and proteolytic degradation, thus maintaining FGF2 function. This suggests that EVs protect soluble factors from degradation and increase their stability and half-life. These results reveal a novel aspect of EV function and suggest EVs as a potential tool for delivering FGF2 in skin healing therapies.

## Introduction

Tissue homeostasis and repair rely on tightly regulated inter-cellular communication tools. Secreted soluble molecules (growth factors, cytokines, chemokines…) are key signaling mediators that can diffuse within the tissue and activate various types of cells to adjust cell behavior upon specific context. The half-life of soluble factors is highly variable and can range from minutes to hours^1^. In vivo, it is determined by parameters such as intrinsic structure and energy, the presence of proteases and binding to other proteins. Degradation occurs rapidly in the extracellular space due to more or less specific proteases, and binding of cytokines to protective proteins on the cell surface or in the extracellular matrix limits the degradation rate and appears to be a major way for soluble molecules to maintain stability and function.

In skin, cutaneous injury is followed by a step-wise series of sequential and overlapping phases such as clotting, inflammatory infiltration, the formation of granulation tissue and re-epithelialization followed by tissue remodeling and wound contraction. Such a multistep process is coordinated by successive waves of soluble mediators (growth factors, cytokines, ECM components) secreted by resident and infiltrating cell types^2,3^. Many studies point to Fibroblast-Growth factor-2 (FGF2 or basic FGF) as a key player during wound healing. FGF2 is found in wound fluid in the early stages after cutaneous injury^4,5^. In murine models, impaired wound healing in aged mice is associated with reduced levels of FGF2^6^ and FGF2-null mice show reduced collagen deposition at the wound site, retardation of re-epithelialization leading to delayed healing^7^. Moreover, it has been shown that local application of FGF2 stimulates tissue repair^8,9^. FGFs act as signal molecules that bind and activate FGF receptors (FGFRs). Activated FGFRs mainly mediate cell survival through a cascade of phosphorylation events, especially involving PI3 kinase/AKT and the RAS/MAP kinase^10,11^. In addition to its mitogenic effect on dermal fibroblast cells (DFs), FGF2 also regulates migration and differentiation as well as vascular angiogenesis^12^.

Given the medical challenge posed by chronic cutaneous wounds in patients with impaired skin repair (diabetic limb ulcers, burned epidermis…), FGF2 is considered a potent therapeutic tool for treatments^13^. The use of FGF2 in clinical settings to improve impaired wound healing, however, is seriously limited due to its short half-life and poor stability in vitro and in vivo. It has long been known that the FGF2 structure is particularly unstable and also subjected to aggregation. Compared to other members of the large FGF family, FGF2 is highly sensitive to pH change and temperature^14,15^. A particular region known as the heparin-binding site has been suggested as the origin of FGF2 instability. A large body of evidence has shown that binding of heparin or heparan sulfates (HS)—in particular heparan sulfate proteoglycans (HSPGs)—stabilizes the FGF2 structure and function via the formation of a ternary complex composed of FGF ligand, FGF receptor and HSPGs^15–17^.

In addition to soluble mediators, cells communicate through the secretion and uptake of a variety of extracellular vesicles (EVs) of different sizes and contents. EVs are composed of a bilipidic membrane and contain numerous molecules such as mRNAs, miRNAs, proteins and metabolites specific to the producing cells. EVs are released either by direct plasma membrane budding or by secretion from multivesicular bodies (MVB), the EVs resulting from the latter process are called exosomes. EVs can act locally on neighboring cells or on distant organs through their circulation in body fluids^18–20^. Some studies have shown that EVs can contain soluble molecules such as cytokines and that EVs could be a route of secretion for cytokines lacking secretion signal peptides^21^. In skin, endogenous EVs participate in tissue homeostasis^22–24^. A large number of studies have reported the therapeutic potential of EVs when applied locally to promote wound repair in mouse models^25^, in particular, EVs isolated from mesenchymal stem cells (MSCs), which promote skin repair through immunoregulatory and regenerative effects^26,27^.

In light of the emerging role of EVs in the secretion and transport of cytokines, and the importance of FGF2 in skin repair, we investigated the interplay between EVs and FGF2. We hypothesized that EVs could participate in intercellular communication during wound repair and interact with FGF2 function in this context. Here we report for the first time that FGF2 binds to the external surface of a subpopulation of EVs. This specific association with EVs potentiates FGF2 signaling, DF proliferation and migration as well as in vivo wound repair. Our results also show that this potentiation effect can be explained by a protective function of this association on FGF2, resulting in increased stability of the cytokine, and thus possibly increased bio-availability in the extracellular space.

## Results

### EVs from FGF2-treated DFs harbor FGF2 on the outer membrane

We mimicked the wound environment by stimulating DFs with FGF2, usually produced in the wounded skin by keratinocytes and immune cells, and investigated whether FGF2 could be associated with produced EVs. EVs were isolated from primary human DFs either unstimulated (CTL-EVs) or following FGF2 treatment (FGF2-EVs) using classical sequential centrifugations^28^. Nanoparticle Tracking Analysis revealed that FGF2 neither influenced the number of particles secreted per cell nor the size distribution of the secreted vesicles (Suppl. Fig. 1A). Western blot analysis confirmed that CTL-EVs and FGF2-EVs contained markers commonly found in EVs^29^, including tetraspanins (CD63, CD81 and CD9), syntenin-1 and TSG101, and were negative for endoplasmic reticulum and cytoplasmic markers, respectively calnexin and actinin-4 (Suppl. Fig. 1B).

**Figure 1:**
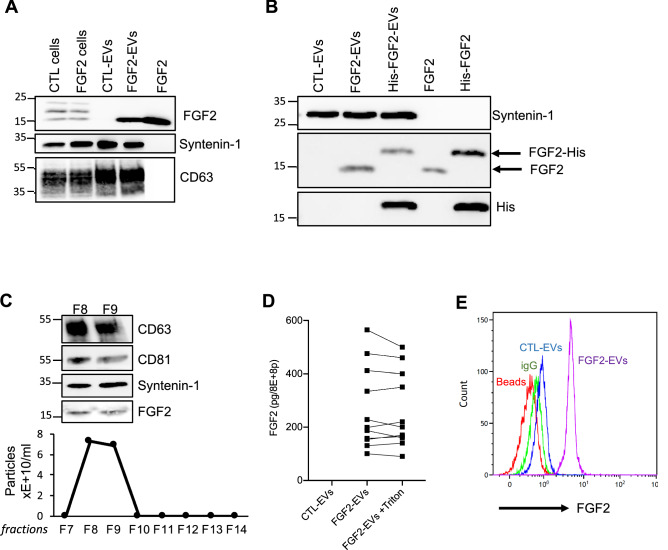
FGF2 is found at the outer surface of EVs secreted by dermal fibroblasts cultured in FGF2-containing medium. (**A**) FGF2, syntenin-1 and CD63 expression in DF cells and EV lysate (20 μg) was examined by western blot. Three isoforms of endogenous FGF2 were present in DF cells while FGF2-EVs contained the low molecular weight FGF2 isoform corresponding to recombinant FGF2. Original blots are shown in Suppl. Information. (**B**) Cells were cultured in the presence of His-tagged FGF2; western blot with 15 μg lysate and using anti-His antibody revealed the presence of His-FGF2 in secreted EVs. A shift in FGF2 size was also observed. Syntenin-1 was used as EV marker. Original blots are shown in Suppl. Information. (**C**) FGF2-EVs were loaded on a SEC column and collected fractions 8 and 9 (3E + 9p) were analyzed by western blot using antibodies for FGF2 and the EV markers CD63, CD81 and syntenin-1. Original blots are shown in Suppl. Information. For quantification, fractions were also analyzed by NTA. (**D**) FGF2 detection in FGF2-EVs by ELISA assay. Intact CTL- and FGF2-EVs (8E + 8p) were directly placed on FGF2 ELISA wells for external surface detection. FGF2-EVs were also treated with 0.1% triton and analyzed to detect internal FGF2. No FGF2 signal was detected in CTL-EVs. (**E**) FGF2 expression on FGF2-EVs was detected by flow cytometry. EVs were coupled to latex beads and labeled with FGF2 antibody before analysis by cytometry. Beads without EVs (beads) and beads labelled with goat IgG antibody (IgG) serve as negative controls. A representative plot is shown.

Using western blot, we searched for the presence of FGF2 in DFs and secreted FGF2-EVs. Endogenous cellular FGF2 isoforms were detected in cellular extracts but absent in CTL-EVs (Fig. 1A). FGF2-treated DFs, however, were found to secrete EVs that containing FGF2, although the molecular size of the recombinant FGF2 used to treat the cells could not be distinguished from one of the endogenous isoforms (Fig. 1A). To validate whether recombinant FGF2 is present in FGF2-EVs, DFs were treated with His-tagged FGF2 and western blot revealing the His tag confirmed that exogenous FGF2 is present in secreted EVs (Fig. 1B). To exclude co-purification of soluble FGF2 together with the EVs by ultracentrifugation, an additional step of purification was performed using size-exclusion chromatography columns, which allow size-based separation between vesicles and soluble proteins. Collected fractions 8 and 9 contain most of the particles and FGF2 associated with the EVs was still detected by western blot (Fig. 1C). Taken together, these results show that DFs cultured in the presence of recombinant FGF2 secrete EVs containing the recombinant FGF2 itself. We next examined whether the FGF2 molecule was located inside the vesicles or at the external membrane of the secreted EVs. FGF2-EVs were assayed in FGF2 Elisa in the presence of triton to allow access to intra-luminal EV content. The detected FGF2 signal was similar in the absence or presence of triton, demonstrating that FGF2 was exclusively located on the outer EV membrane (Fig. 1D). The FGF2 external presence was confirmed by flow cytometry. CTL-EVs and FGF2-EVs were incubated with latex beads to allow binding, followed by labeling with anti-FGF2 antibody and fluorescent secondary antibody. Figure 1E shows the detection of FGF2 at the surface of the FGF2-EVs.

### Purified CTL-EVs can bind FGF2

**Figure 2:**
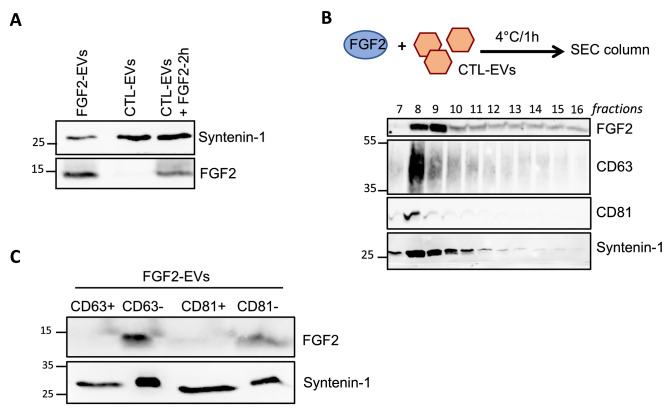
A CD63/CD81-negative subpopulation of EVs binds FGF2. (**A**) Cell-independent binding of FGF2 to DF-EVs. Conditioned medium from amplified CTL cells was transferred into new dishes. FGF2 (100 ng/ml) was added for 2 h at 37 °C and the medium was further processed for EV isolation. FGF2 expression in EVs (15 μg) was determined by western blot and compared to CTL- and FGF2-EVs. (**B**) Binding of FGF2 to purified CTL-EVs. FGF2 (100 ng/ml) was incubated with purified CTL-EVs (1E + 10p) for 1 h on ice before insertion into the SEC column. One hundred microliters from 200 μl of each collected fraction were analyzed by western blot with FGF2, CD63 and syntenin-1 antibodies. (**C**) Immuno-isolation of FGF2-EVs (1E + 10p) with beads coupled with anti-CD63 or anti-CD81 antibody. Positive (total amount) and negative (1:5 (v/v) of the flow-through) fractions were analyzed by western blot for FGF2 and syntenin-1 expression. Representative blots are shown. Original blots are shown in Suppl. Information. Validation of the immunoprecipitation is shown in Suppl. Fig. 1D.

Our observation—that secreted EVs carry exogenous FGF2—leads to questions about the underlying molecular mechanism. We thus examined whether binding of FGF2 was cell-dependent. To do so, we transferred DF medium from a 48 h culture (containing CTL-EVs) to new culture dishes (without cells) and added 100 ng/ml of FGF2 for an extra 2 h at 37 °C before EV purification. Analysis of these EVs revealed the presence of associated FGF2 in the absence of cells (Fig. 2A). We further confirmed FGF2’s capacity to bind purified EVs by incubating purified CTL-EVs with soluble FGF2 for 1 h on ice. A size exclusion chromatography (SEC) column was then used to eliminate unbound FGF2 and fractions were analyzed by western blot. A strong FGF2 signal was observed in fractions containing EVs detected by syntenin-1 and CD63 labeling (Fig. 2B).

### FGF2 binds to CD63^neg^CD81^neg^-EVs

Interestingly, analysis of EVs after SEC column (Fig. 2B) revealed that fraction 9 was characterized by the presence of syntenin-1-positive EVs with a high FGF2 signal but low CD63 and CD81 signals. To explore the hypothesis that FGF2 could possibly be predominantly bound to CD63-negative (CD63^neg^) vesicles, FGF2-EVs were analyzed after immune-separation of CD63-positive (CD63^pos^) and CD63^neg^ fractions using CD63 antibody-coated beads. Western blot analysis confirmed the presence of syntenin-1^pos^/CD63^neg^ vesicles. FGF2 signal was found exclusively in the CD63^neg^ fraction (Fig. 2C). Similarly, we used CD81 antibody-coated beads to isolate CD81^pos^ vesicles from FGF2-EVs and examined the presence of FGF2 in CD81^pos^ and CD81^neg^ fractions by western blot. FGF2 was detected only in the syntenin-1^pos^/CD81^neg^ fraction (Fig. 2C). We confirmed the efficiency of the commercial CD63 or CD81 antibody-coated beads to immune separate positive and negative fractions (Suppl. Fig. 1C).

To identify putative FGF2-binding proteins on the EV surface, we performed proteomic analysis after immuno-separation using beads coated with the FGF2 antibody to isolate FGF2^pos^ vesicles from FGF2^neg^ EVs (Suppl. Fig. 2A). CTL-EVs were used as control and also incubated with anti-FGF2-coated beads. Proteomic analysis was done using mass spectrometry on the following four experimental groups: CTL-EVs pulldown and flow through (annotated PD-CTL-EVs and FT-CTL-EVs, respectively) and FGF2-EVs pulldown and flow through (annotated PD-FGF2-EVs and FT-FGF2-EVs, respectively) were analyzed (Suppl. Fig. 2A). Qualitative protein analysis revealed a total of 1029 identified proteins. A comparison with the Top100 most described proteins in EVs reported in the ExoCarta site revealed that 89 out of the 100 EV-related proteins were found in the analysis (Suppl. Fig. 2B-a). Quantitative analysis was performed to identify proteins specifically abundant in FGF2-binding EVs, by comparing the anti-FGF2 PD fraction with the FT from the FGF2-EVs. To exclude non-specific binding revealed by immunoprecipitation with anti-FGF2 on the CTL-EVs, the anti-FGF2 PD fraction from the FGF2-EVs was also compared to the anti-FGF2 PD fraction from the CTL-EVs (Suppl. Fig. 2B-b). Altogether, 824 proteins were significantly more expressed-or unique- in the PD-FGF2-EVs fraction either compared to either FT-FGF2-EVs or PD-CTL-EVs (Suppl. File 1). Among these 824 proteins, 391 proteins were found to be correlated up in both comparisons. These proteins characterize FGF2-binding vesicles and should contain FGF2-binding proteins. The analysis revealed several proteins previously known to bind FGF2 including Glypican-1 (GPC1), chondroitin sulfate proteoglycan-4 (CSPG4 or NG2), or latent transforming growth factor beta binding-2 (LTBP2). In addition, thrombospondin-1 and 2, which can bind FGF2, were detected only when we compared PD-FGF2-EVs and FT-FGF2-EVs. Confirmation of the presence of GPC1, CSPG-4 and thrombospondin-1/2 in FGF2-EVs was done by western blot (Suppl. Fig. 2C). These results revealed that DF-EVs harbor several FGF2 binding proteins on the outer surface that could participate in FGF2 binding.

### Binding to EVs protects FGF2 from degradation

**Figure 3:**
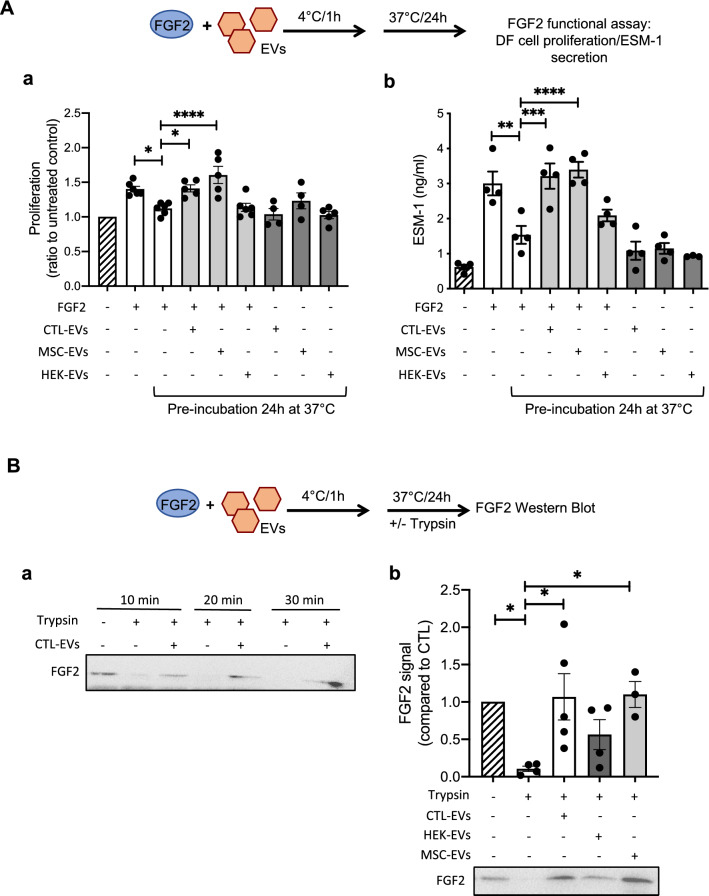
EV-bound FGF2 is protected from degradation. (**A**) Effect of EV binding on FGF2 thermal stability. FGF2 (10 ng/ml), alone or after incubation with EVs (8E + 8p) from different cell types for 1 h on ice, was placed at 37 °C for 24 h to challenge FGF2 thermal stability. Residual FGF2 activity was determined by (**a**) proliferation assay by BrDU incorporation and (**b**) ELISA assay to measure ESM-1 secretion. EVs from DF, MSC, HEK cells were tested. Results are mean ± SE of four independent experiments. Statistical significance was determined by one-way ANOVA (Dunnett’s multiple test), *< 0.05, **< 0.01, ***< 0.001, ****< 0.0001. (**B**) Effect of EV binding on FGF2 degradation by trypsin. FGF2 (10 ng), alone or after incubation with EVs from DF, MSC or HEK cells (8E + 8p) for 1 h on ice, was held at 37 °C with trypsin/EDTA (0.05%, diluted 1:2 v/v in 25 μl) for different time periods before loading on western blot to assess residual FGF2 protein. (**a**) Representative blot with CTL-EVs (**b**) Representative blot and quantification of FGF2 signal obtained after 10 min incubation with EVs from DF, MSC or HEK cells. Results are mean ± SE of 3–5 independent experiments. Statistical significance was determined by one-way ANOVA (Dunnett’s multiple test), *< 0.05. Original blots are shown in Suppl. Information.

The discovery that soluble FGF2 anchors to the EV surface suggests that EVs could serve as FGF2 carrier in the extracellular space. As FGF2 is a particularly unstable cytokine with a short-half-life, we hypothesized that binding to EVs could stabilize the FGF2 molecule and delay its denaturation. The functional integrity of the FGF2 molecule was assayed by two functional assays, a DF proliferation assay and by measuring the ESM-1 secretion^30^. When placed at 37 °C for 24 h, soluble FGF2 lost its activity as detected by reduced proliferation and ESM-1 secretion (Fig. 3A). When soluble FGF2 was pre-incubated for 1 h on ice with CTL-EVs before incubation at 37 °C for 24 h, FGF2 activity was maintained. Proliferation and ESM-1 secretion were at similar levels compared to fresh FGF2, which served as a positive control, demonstrating than DF-EVs protect FGF2 from natural instability under physiological temperature (Fig. 3A). We next questioned whether other types of EVs could present this same ability to protect FGF2. We examined other cell types that are cultured with FGF2 such as mesenchymal stem cells (MSCs) and pluripotent stem cells (iPSCs). MSCs and iPSCs are routinely cultured with 10 ng/ml and 100 ng/ml FGF2, respectively and secreted EVs were purified and analyzed by western blot. As with DFs, FGF2 was found to be present in secreted EVs from iPSCs and MSCs. MSC-EVs display less FGF2 in accordance with the lower concentration of FGF2 used in the culture medium (Suppl. Fig. 3). To test whether FGF2 binding to EVs is universal, EVs secreted from HEK cells and MSCs were tested in the same assays. While MSC-EVs protected FGF2 to a similar extent as CTL-EVs, HEK-EVs were not able to prevent FGF2 inactivation (Fig. 3A). This observation could be explained by the lack of GSPG4 and thrombospondin on HEK-EVs, whereas both are abundant on MSC-EVs (Suppl. Fig. 2C). These results suggest that specific EVs, such as EVs derived from DFs or MSCs, have the capacity to protect FGF2.

We next evaluated the potential of EVs to protect FGF2 from protease activity and we used the unspecific protease trypsin. Trypsin induces rapid degradation of soluble FGF2 as shown by the disappearance of the intact form of FGF2 detected by western blot (Fig. 3B-a). Pre-incubation with CTL-EVs prevented trypsin-induced degradation of FGF2 even after 30 min (Fig. 3B-a). MSC-EVs showed similar protection activity while HEK-EVs could only partially maintain the FGF2 molecule (Fig. 3B-b).

### EVs produced by FGF2-stimulated DFs activate survival signaling pathways

**Figure 4:**
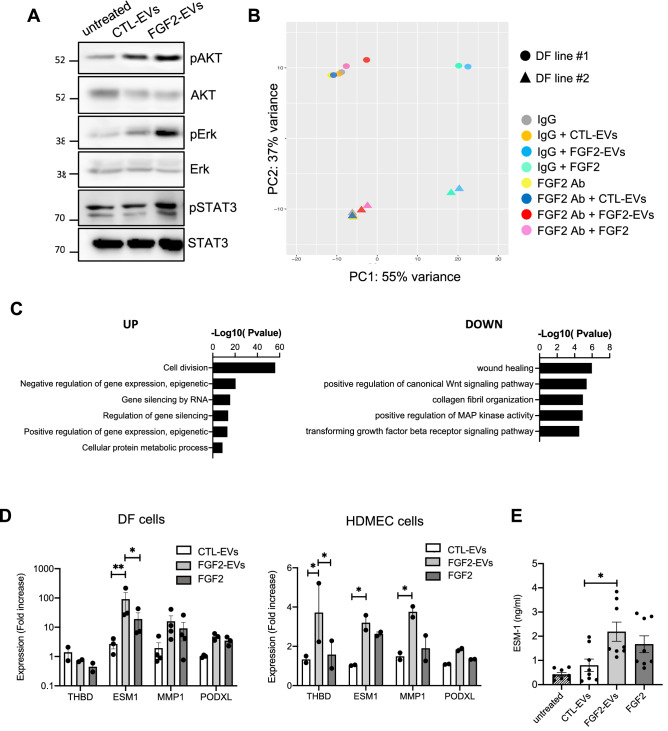
Activated pathways by FGF2-EVs in DF cells. (**A**) ERK, AKT and STAT pathways are activated by FGF2-EVs. DF cells were treated with CTL-EVs or FGF2-EVs (1E + 9p/ml) for 10 min and activation of ERK, AKT and STAT3 was revealed with the detection of phosphorylation forms by western blot from 20 μg proteins. Total forms of ERK, AKT and STAT3 proteins were also detected to assess total amount of protein. Representative blots are shown. Original blots are shown in Suppl. Information. (**B**,**C**) RNA-seq analysis after FGF2-EV treatment. Two DF cell lines were treated with PBS, CTL-EVs or FGF2-EVs (2E + 10p) or FGF2 (100 ng/ml) for 24 h, following which RNA-seq was performed. Additional samples were pre-treated with neutralizing FGF2 antibody or IgG control 1 h before EV treatment. (**B**) PCA plot (**C**) GO terms of upregulated and downregulated genes between CTL-EVs and FGF2-EVs. (**D**) Validation by RT-PCR. DF cells and HDMEC cells were treated with PBS, CTL-EVs, FGF2-EVs or recombinant FGF2 for 24 h and real-time PCR was performed with primers specific to THBD, ESM1, MMP1 and PODXL genes. The data are mean ± SE of 2–4 independent experiments. Statistical significance was determined by two-way ANOVA (multiple comparison test with Tukey correction), *< 0.05, **< 0.01. (**E**) Concentration of extracellular ESM-1 protein in conditioned medium of treated DF cells was determined by ELISA. The data are mean ± SE of four independent experiments. Statistical significance was determined by ordinary one-way ANOVA (multiple comparison test with Tukey correction). *< 0.05.

To investigate the effect of FGF2-EVs on recipient cells more precisely, the classical PI3K/AKT, MAPK/ERK and STAT pathways were examined. DFs were stimulated with CTL-EVs and FGF2-EVs and phosphorylated forms of AKT, ERK and STAT3 kinases were detected by western blot. Treatment with FGF2-EVs led to a robust phosphorylation of ERK and STAT3, which was not found with CTL-EVs, and, to a lower extent, of AKT (Fig. 4A). Next we performed RNA-seq analysis after EV treatment to examine modulated genes in DFs, potentially by PI3K/AKT and MAPK/ERK pathways. Twenty-four hours after stimulation with CTL-EVs or FGF2-EVs, RNA was extracted, and used for RNA-seq. Principal component analysis (PCA) showed a clear gene expression difference between CTL-EVs and FGF2-EVs, on the PC1 axis that represents 55% variance (Fig. 4B). Analysis revealed a list of genes upregulated or downregulated by FGF2-EVs, compared to CTL-EVs (Suppl. File 2). Analysis of significant enriched Gene Ontology (GO) biological processes terms (Fig. 4C) showed that genes downregulated upon FGF2-EVs exposure are enriched with genes related to wound healing and ECM as well as Wnt and TGF-β pathways, suggesting that FGF2-EVs could also participate in skin remodeling also by negatively regulating several pathways.

For upregulated genes, although cell division-related functions were the most significant GO terms, the most upregulated genes by FGF2-EVs, in terms of fold increase, were the four genes *thrombomodulin* (THBD), *endothelial cell specific molecule-1* (ESM-1), *matrix metalloprotease-1* (MMP-1) and *podocalyxin-like* (PODXL) (Suppl. Fig. 4A and Suppl. File 2). When comparing our list of genes activated by FGF2-EVs or FGF2 with that of dermal fibroblasts stimulated with FGF2 performed by Kashpur et al.^31^, we found that 117 of the 291 genes are commonly activated more than 1.5-fold in both studies and that the list of top 15 genes in both studies contains 13 common genes (Suppl. File 2). Of note, among these, the most activated genes in our study ESM-1 and MMP-1 are also found in other studies reporting FGF2-induced genes^32,33^. Activation of three of those four genes by FGF2-EVs was confirmed by RT-qPCR in DFs (Fig. 4D). Interestingly, THBD and ESM-1/endocan have, to date, been mostly described in endothelial cells^34,35^. Endothelial cells also participate in skin repair by remodeling the vascular network. Thus, we also examined the effect of DF-EVs on human dermal microvascular endothelial cells (HDMEC cells) and observed similar effects on gene activation (Fig. 4D).

Intriguingly, ESM-1/endocan was the most highly upregulated gene by FGF2-EVs in DF cells. Using an ELISA assay, increased secretion of extracellular ESM-1 was detected after FGF2-EVs treatment in DFs (Fig. 4E). Moreover, we found that soluble FGF2 increases ESM-1 expression and secretion in DFs significantly—although not as much as FGF2-EVs at both RNA and protein levels, suggesting that FGF2-EVs may be more potent than soluble FGF2 (Fig. 4C–E).

Altogether, these results show that FGF2-EVs have the capacity to induce a range of cellular responses via gene modulation in at least two skin cell types, fibroblasts and endothelial cells.

### FGF2-EVs induce DF proliferation, migration in vitro and accelerate wound repair in vivo

**Figure 5:**
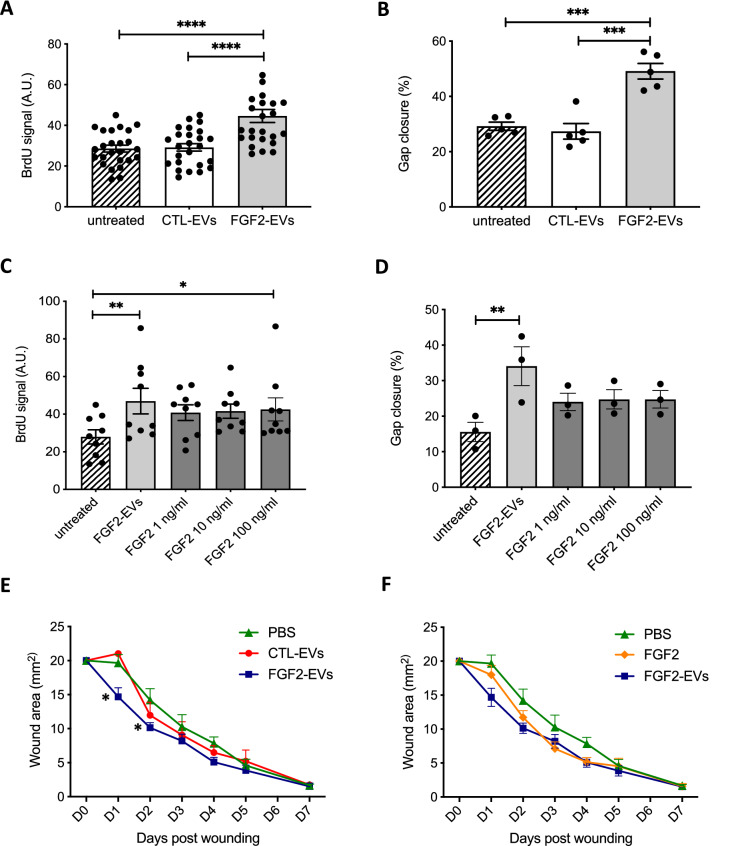
FGF2-EVs activate fibroblast functions. The effect of CTL-EVs and FGF2-EVs was assessed on DF proliferation (**A**,**C**) and migration (**B**,**D**). DF proliferation was assayed by BrDU incorporation 48 h after treatment with PBS (untreated), CTL-EVs or FGF2-EVs. Fibroblast migration was assayed by wound gap closure assay. PBS, CTL-EVs or FGF2-EVs were added immediately after insert removal; gap closure was measured 48 h later. (**A**,**B**) Statistical significance was determined by one-way ANOVA, ****< 0.0001, ***< 0.001. (**C**,**D**). The effect of FGF2-EVs was compared with different concentrations of recombinant FGF2 from 1 to 100 ng/ml in proliferation and migration assays. The data are mean ± SE of 3–4 independent experiments. Statistical significance was determined by one-way ANOVA **< 0.01, *≤ 0.05. (**E**,**F**) The effect of FGF2-EVs was assayed in vivo through a cutaneous wound assay in mice. Following wound (D0), EVs (1.5E + 9p) in a volume of 125 μl were given to each mouse, of which 25 μl were topically applied on the wound and 100 μl were injected in four locations surrounding the wound. Pictures were taken daily from day (D) 0 to D5 as well as on D7 and used for wound area quantification. Results are mean ± SE from one representative experiment with n = 2 (non-injected), n = 3 (PBS) and n = 5 for CTL-EVs and FGF2-EVs. Statistical significance was determined by two-way ANOVA with Tukey’s correction of multiple comparison: significance was found on D1 for FGF2-EVs compared to PBS (*< 0.05) and CTL-EVs (**< 0.005) and on D2 for FGF2-EVs compared to PBS (*< 0.05).

To examine the potential of FGF2-EVs on surrounding cells, DFs were treated with FGF2-EVs and functional responses involved in wound repair such as proliferation and migration were assessed. As RNA-seq analysis revealed a large number of genes related to cell cycle activated by FGF2-EVs, we examined DF proliferation using a BrdU incorporation assay. Treatment of DFs with FGF2-EVs enhanced their proliferation by 52%, whereas the CTL-EVs only led to a 7% increase (Fig. 5A and Suppl. Fig. 5A). To decipher what activated pathways are regulating cell proliferation, selective chemical inhibitors of pathways were tested in FGF2-EV-induced cell proliferation. Inhibitors of the PI3K/AKT pathway (GDC-0890) and of ERK (U0126) had no effect on proliferation while inhibition of the STAT pathway with SH-4-54 significantly blocked the effect of FGF2-EVs on proliferation (Suppl. Fig. 4B).

In vitro DF migration tested by wound healing IBIDI assay was also enhanced by FGF2-EVs treatment whereas EVs from untreated DFs had no effect (Fig. 5B). To further evaluate the potency of FGF2-EVs, we compared FGF2-EVs with various concentrations of soluble FGF2. The results revealed that FGF2-EVs are at least as potent as soluble FGF2 for cell proliferation (Fig. 5C). Similar results were also observed on cell migration, although these were not statistically significant (Fig. 5D). Finally, we examined endothelial cell proliferation and found that FGF2-EVs increased cell proliferation to a similar level as VEGF used as a positive control (Suppl. Fig. 4C).

Fibroblast proliferation and migration are key events during skin repair. To examine whether FGF2-EVs would enhance in vivo wound healing, FGF2-EVs were injected on day 0 around the margin of a 5 mm-punch wound in young adult mice. This unique injection of FGF2-EVs significantly improved the initial steps of wound healing on day 1 (n = 5 per group, two independent experiments) (Fig. 5E). Comparison with soluble FGF2 shows a faster skin recovery on day 1 with FGF2-EVs than recombinant FGF2 (Fig. 5F).

**Figure 6:**
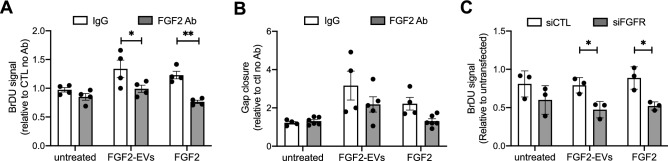
Effect of FGF2 inhibition on FGF2-EV activity. FGF2 effects on DF proliferation (**A**,**C**) and migration (**B**) were inhibited by neutralizing FGF2 antibody (**A**,**B**) or siRNA (**C**). FGF2-EVs were incubated with neutralizing FGF2 antibody or IgG control for 30 min prior to being added to the DF cells. The data are mean ± SE of three or four independent experiments. Statistical significance was determined by two-way ANOVA, *< 0.05, **< 0.01.

Collectively, these results suggest that FGF2-stimulated fibroblasts secrete modified EVs that are tissue mediators that could participate in dermis remodeling during skin healing. They also, however, lead to the question of whether the effects of FGF2-EVs described previously are due to surface FGF2. To examine the role of external FGF2 we used two inhibitory tools for FGF2 signaling, neutralizing FGF2 antibody and siRNA against FGF receptors. FGF2-EVs were incubated with FGF2 antibody 30 min before applying to DFs in proliferation and migration assays. Blocking FGF2 inhibited the effect of FGF2-EVs on cell proliferation (Fig. 6A) and migration (Fig. 6B). Similarly, siRNA knocking down FGF2 receptors (FGFR1-3, Suppl. Fig. 5B) in DFs prevented FGF2-EV increasing proliferation to a similar extent as it prevented soluble FGF2-induced proliferation (Fig. 6C). In addition, the effect of neutralizing FGF2 antibody on FGF2-EV-induced gene activation was assessed in RNA-seq analysis. The PCA profile revealed that neutralizing FGF2 reduced FGF2-EV-induced gene expression change dramatically, to a level that is comparable to CTRL-EV treatment (Fig. 4B). Moreover, the PCA profile revealed high similarities in genes induced by FGF2-EVs and soluble FGF2, confirming that surface FGF2 on FGF2-EVs was responsible for most of the effect induced by FGF2-EVs. Comparing the FGF2-EVs profile with the FGF2 profile, only three genes were differently regulated: ESM-1, PODXL and ARHGAP28, which were all significantly more activated by FGF2-EVs. We confirmed that ESM-1 was significantly more activated by FGF2-EVs than FGF2 at both mRNA and protein levels in DFs (Fig. 4D,E). These results show that FGF2 on the EV surface mediates most gene activation by FGF2-EVs in fibroblasts and that the FGF2 pathway is at least as potent when presented on the EV surface as soluble FGF2.

## Discussion

FGF2 controls key cellular events during development and in various adult tissues such as cell proliferation and cell migration. Its well-demonstrated beneficial role on wound healing has been limited by its short half-life, mainly due to intrinsic structural instability and high sensitivity to temperature and pH level. In light of FGF2’s beneficial effect on wound healing, in recent years efforts have been made to stabilize FGF2 through the development of mutant FGF2, chemically modified FGF2, stabilizing formulations and delivery systems for clinical applications^14^. In fact, FGF2 bioavailability relies on a large number of protecting molecules such as membrane-associated heparan sulfate proteoglycans (HPGP) and matrix proteins that are able to bind and stabilize FGF2^36–39^. The importance of FGF2 binding to heparan sulfates for its stability was demonstrated by several in vitro studies that revealed the protective role of heparin towards FGF2 against heat-, acid- and proteases-induced degradation^15,36,40^.

In this study, we uncovered a novel mechanism regulating FGF2 stability. We demonstrated that FGF2 can spontaneously bind to the outer EV surface in extracellular medium but also acellularly when incubated in the presence of isolated EVs. We further showed that this binding protects FGF2 from degradation.

Intriguingly, the mechanism of FGF2 secretion is still being debated as the FGF2 sequence does not include a classical export signal. Contradictory studies have proposed or excluded EVs as a way of FGF2 secretion^41,42^. Nevertheless, studies have reported the localization of endogenous FGF2 in EVs secreted by hepatoma cells^42^, astrocytes^43^, neurons^44^ skeletal muscle cells^45^ and bone marrow cells^46^. Our observation that endogenous FGF2 expressed in resting DF cells is absent in secreted EVs suggests that FGF2 is not secreted in DFs via EVs, at least in the absence of stimuli. Concerning extracellular FGF2, which is known to be internalized into the endosomal process after signaling, we could not detect exogenous FGF2 in EVs after a short stimulation (Suppl. Fig. 5C), excluding FGF2 processing in the endosomal and MVB route after internalization, at least under those particular experimental conditions.

We demonstrated here that EVs from DFs, and also MSC cells, can bind FGF2 on their surface, either in cell culture or in a test tube in the absence of cells. Previous studies have reported the presence of cytokines or soluble factors in- or on- EVs, suggesting that EVs function as cargo ships transporting these molecules to distant areas for signaling^21^. The cytokines IL-18^47^, TNF-alpha^48^, TGF-β^49,50^ and IL-8^51^ are present on the EV outer surface. Recently, the comprehensive study by Fitzgerald et al. revealed that the association of cytokines with EVs is widely found in several types of immune cells tested and the ratio between free and EV-associated cytokines is highly variable depending on cell type and stimuli^52^. Moreover, they examined the localization of EV-associated cytokines and found cell- and cytokine-specific distribution in the lumen or on the outer EV surface^52^. Another seminal study by Ko et al. reported that secreted VEGF can be found under free soluble form together with an EV-associated form, the latter being the specific VEGF_189_ isoform. Furthermore, EV binding protects VEGF from therapeutic inhibitory antibody conferring resistance^53^. We have also recently observed that the cytokine CSF-1 is both present on the surface of EVs and as a soluble form in the conditioned medium of some tumor cell lines, and that EV-associated CSF1 and recombinant CSF1 induce different gene expression signatures in monocytes^54^. In sum, the above-mentioned studies reveal that EV-bound cytokines are a common phenomenon in biology.

Here we further demonstrated that FGF2 binding to EV surface can occur in the absence of cells, suggesting that soluble factors secreted in the extracellular space could be caught by EVs secreted by other cell types. Moreover, we show that FGF2 binding is EV-specific, as FGF2 binds to a distinct EV population, characterized by the absence of CD63/CD81 markers. These unexpected results raise questions about the nature of FGF2-binding EVs. EVs obtained by ultracentrifugation are highly heterogenous. Bulk populations and classical tetraspanin markers are not constitutively expressed on all subsets^55,56^. CD63 is considered enriched in small EVs of late endosomal origin, whereas CD81 is likely more associated with plasma membrane-derived EVs^57^ and little is known about vesicles lacking those markers. One can speculate that FGF2-binding EVs might not be of endosomal origin but rather microvesicles formed by membrane budding, although a study analyzing EVs by density gradient revealed that endosomal ESCRT-positive EVs (expressing the Alix marker) were distinct from CD63-positive EVs, suggesting the existence of CD63-negative endosomal exosomes^58^.

Second, comparison between EVs from different cell types revealed that EVs from DFs and MSCs similarly bind and protect FGF2 while EVs from HEK cells protected FGF2 much less efficiently, again suggesting a highly specific binding mechanism mediated by FGF2-binding molecules located on the EV surface. In search of these, our analysis using mass spectrometry revealed several candidate proteins for FGF2 binding on the EV surface. Among FGF2-binding proteins revealed by the analysis, we noticed trombospondin-1 and -2 and CSGP4, which are of particular interest as they were not detected on HEK-EVs. Thrombospondin is a matrix protein that binds several growth factors, including FGF2^59,60^ and CSPG4 (or CSPG4/N2) is a transmembrane chondroitin sulfate proteoglycan that can interact with PDGF and FGF2 in the presence or absence of GAGs and promote FGF2 signaling^61^. Other studies have proposed proteoglycans for TGF-β binding on cancer EVs via binding to HS chains such as betaglycan^49,50^. Further work is therefore needed to fully characterize molecularly the FGF2 binding to EVs. For example, it would be interesting in future studies to overexpress thrombospondin and/or CSPG4 in HEK cells or to inactivate them in fibroblasts in order to evaluate the respective effects on the capacity of HEK-EVs to bind and protect FGF2.

We showed that EV-bound FGF2 is fully functional and activates classical signaling pathways similarly to soluble FGF2 in DFs, leading to similar gene activation, and increases DF proliferation and migration. Nevertheless, we found several noticeable differences: (i) ESM-1 was the only gene more significantly activated with EV-bound FGF2, (ii) DF migration and proliferation were slightly increased with EV-bound FGF2 and (iii) in vivo, EV-bound FGF2 demonstrated more potency vis-à-vis accelerating wound closure. Our results are in line with previous studies reporting differential cytokine function when bound to EVs^49,50,53,62^ and underlines the importance of considering the EV-bound fraction when studying cytokine function.

Our study further proposes a stabilization mechanism that could explain increased FGF2 function. We established that FGF2 binding to EVs becomes protected from either heat- or trypsin-induced degradation. We further found that, in vitro at 37 °C, FGF2 ceased being active within one day, consistently with previous reports^63^. Pre-incubation with DF-EVs or MSC-EVs prevented the decrease of FGF2 function, maintaining FGF2 activity to the same extent as intact FGF2. Binding to EVs was also efficient enough to prevent trypsin-induced proteolytic action, suggesting that EV-bound FGF2 molecules could be protected from environmental proteases while maintaining their function. This is, to our knowledge, the first study showing increased cytokine stability and protection when bound to EVs. Accordingly, EVs can now also be viewed as potent regulators of cytokines spatial and temporal activity. FGF2 stabilization may explain our observation that FGF2-EVs accelerate wound closure in vivo. The effect is observed mainly on day 1 or 2 after wounding, suggesting that FGF2-EVs rather promote the early reepithelization step. Complete histological analyses of the skin at different time points, dose response experiments, and additional mouse models would bring further insights into the benefits of EV-bound FGF2 for skin repair.

Altogether, our results support an emerging paradigm for EV function: as carriers for and protectors of soluble molecules. This may also open new directions for using EVs in efforts to deliver soluble molecules with long-lasting effects in the context of tissue repair.

## Materials and methods

### Cell culture

Primary human dermal fibroblasts (DFs) were isolated from skin samples obtained from abdominal surgeries conducted in the Division of Plastic and Reconstructive Surgery, Hôpital Saint Louis, Paris, following receipt of informed consent from the patients. Samples were washed in HBSS and fat tissue was removed. To separate the dermis and epidermis, tissue pieces were incubated overnight at 4 °C in 0.5% Dispase II. DFs were isolated as previously described, with minor modifications^64^. The dermis was finely chopped and incubated in 3 mg/ml Collagenase IV for 2 h at 37 °C under agitation. Following homogenization by pipetting, the obtained cell solution was filtered through a 70 μm filter and centrifuged. Cells were seeded at a density of approximately 20,000 cells/cm^2^ in high glucose-DMEM containing 10% FCS, l-glutamine and 1% penicillin/streptomycin (DF medium) and expanded. DFs isolated from four different donors were used: two for EV purifications and two for recipient cells.

Other cells used in this study included MSCs (ThermoFisher) cultured in low glucose-DMEM containing 10% FCS, l-glutamine and 1% penicillin/streptomycin supplemented with FGF2 (10 ng/ml, Miltenyi Biotec), HEK-293T cells cultured in high glucose-DMEM containing 10% FCS, l-glutamine and 1% penicillin/streptomycin and a normal human iPSC line^65^ cultured in serum free E6 medium (ThermoFisher) supplemented with FGF2 (100 ng/ml).

### Purification of secreted extracellular vesicles (EVs)

EVs were purified from DFs as previously described^28^. Briefly, DFs were seeded in 150 mm dishes, at a density of 5E + 5 cells/dish, in DF medium. Five days later, the medium was changed to a DF medium containing 10% EV-depleted FCS (obtained by 18 h of ultracentrifugation of DMEM + 20% FCS at 100,000 rcf in a Type 45Ti rotor). For FGF2 treatment, FGF2 was added at a final concentration of 100 ng/ml (Miltenyi Biotec). Forty-eight hours later, culture medium was taken for EV purification by sequential centrifugations: 300 rcf for 10 min, 2,000 rcf for 20 min, 10,000 rcf for 30 min in a table-top centrifuge, then 100,000 rcf for 90 min (with Type 45Ti rotor, Optima-80 centrifuge, Beckman). The pellet enriched in EVs was resuspended in PBS and ultracentrifuged again at 100,000 rcf for 90 min. Following this, the EV pellet was resuspended in PBS, aliquoted and frozen at − 80 °C. In some experiments (Fig. 1C and confirmation of results presented in Figs. 1D and 5A), further separation was performed by inserting the EV pellet (100 μl) into a size exclusion column according to manufacturer’s instruction (qEVsingle legacy 70 nm columns, IZON) and 200 μl fractions were collected and frozen at − 80 °C. EVs from other cell types were obtained following a similar protocol using EV-depleted serum.

### Nano-particle tracking analysis (NTA)

DF-EV size and concentration measurements were performed by NTA using a NanoSight LM14 (Malvern Instruments) equipped with a sample chamber, containing a 640 nm laser. Samples were injected into the sample chamber with sterile syringes. All measurements were performed at room temperature (RT) and, for each sample, two dilutions with five videos of 60 secs per dilution were analyzed using the NTA 3.1 software.

### Western blot

EV proteins were extracted by incubation with 2% SDS for 15 min at RT. Cell proteins were extracted in RIPA buffer supplemented with Protease and Phosphatase Inhibitor Cocktail EDTA-Free (Thermo Fisher Scientific) for 15 min on ice and centrifuged for 15 min at 4 °C at 15,000 rcf to remove cell debris. Supernatant was collected and protein assays were performed using a Pierce BCA Protein Assay kit (Thermo Fisher Scientific). Fifteen to 20 μg of total protein—or 0.5–5E + 9 particles (p)—were resuspended in a 4 × LDS buffer (Thermo Fisher Scientific) and a 10 × sample reducing agent (DTT, Thermo Fisher Scientific) was added, except for CD9, CD81 and CD63 for which the reducing agent was omitted. Samples were boiled 10 min at 70 °C, loaded on SDS-PAGE gel and transferred to nitrocellulose membrane using the semi-dry method. Membranes were blocked with 5% BSA for phospho-proteins or 5% non-fat milk for the other proteins in Tris-Buffered Saline (TBS) with 0.2% Tween (TBS-T) before primary antibody incubation overnight at 4 °C. In some cases, blots were cut before hybridization with different antibodies. Antibodies used are: CD63 (#556019, 1:1000, BD Biosciences), CD81 (#166029, 1:1000, Santa Cruz Biotechnology), actin (#1615, 1:200, Santa Cruz), calnexin (#11397, 1:100 Santa Cruz Biotechnology), actinin-4 (#101669, 1:1000, Genetex), CD9 (#CBL-162, 1:1000, Sigma), syntenin-1 (#133267, 1:2000, Abcam), TSG101 (Clone 51/TSG101, 1:1000, BD Biosciences), FGF2 (AF-233-NA, 0.1 μg/ml, R&D Systems) phosphorylated forms and total forms of AKT (#4060, 1:1000 and #9272, 1:2000, Cell signaling Technology), ERK (#M8159, 1:5000 and #06182, 1:50,000, Sigma), STAT3 (Ser727 residue, #9134, 1:1000 and #4904, 1:2000, Cell Signaling Technology), CSPG4 (#139406, 1:1000, Abcam), thrombospondin (#59887, 1:200, Santa Cruz Biotechnology), glypican-1 (#226855, 1:1000, Abcam) and FGFR1, FGFR2 and FGFR3 (#9740, #11835 and #4574 respectively, 1:1000, Cell Signaling Technology). Secondary antibodies (Cell signaling Technology) were added for 1 h at room temperature detected by chemiluminescence using Clarity Western ECL Substrate (Bio-Rad) on a gel imaging system (ImageQuant LAS 4000, Bio-Rad).

### Enzyme-linked immunosorbent assay (ELISA)

FGF2 presence in DF-EVs was determined using an ELISA DuoSet kit according to the manufacturer’s instructions (R&D Systems). EVs were tested untreated or after Triton X-100 pre-treatment (1% for 30 min on ice) to permeabilize the EVs. ESM-1 concentration in the condition medium of DF cells treated for 24 h with FGF2 (10 ng/ml) or EVs (1E + 10p) was measured by Elisa according to the manufacturer’s instructions (Lunginnov).

### Flow cytometry

Flow cytometry was performed on EVs bound to latex beads based on published protocol^66^. Briefly, EVs (3E + 9p) were incubated with 10 μl latex beads (aldehyde/sulfate, 4% w/v, 4 μm, ThermoFisher) and continuously rotated overnight at 4 °C. Glycine (100 mM) was added and beads were further incubated for 30 min. Beads were washed three times with PBS/0.5% BSA (centrifugation 3 min, 4000 rpm) and blocked with 100 μl PBS/2.5%BSA for 30 min. Beads were incubated for 30 min on ice with primary goat FGF2 antibody (AF233, R&D Systems) or goat IgG control (AB108C, R&D Systems). Beads were washed with PBS and incubated in 100 μl for 30 min on ice with secondary Alexa Fluor 488 donkey anti-goat antibody (1:500, Invitrogen, ThermoFisher). After wash, beads were resuspended in 800 μl and analyzed on a FC500 (Beckman coulter) flow cytometer with CXP software.

### Immuno-isolation

Immuno-isolation of CD63 or CD81-positive EVs was performed with CD63- or CD81-coupled magnetic beads (Thermo Fisher Scientific) according to the manufacturer’s instructions. Briefly, FGF2-EVs (1E + 10p) were mixed with beads in a total volume of 500 μl and continuously rotated overnight at 4 °C. The flow-through was collected and beads were washed three times with PBS/0.1% BSA. Bead-bound EVs were lysed in 15 μl RIPA buffer supplemented with protease inhibitors (Thermo Fisher Scientific) for 15 min at 4 °C before placing tubes on a magnet to collect the supernatant containing protein lysate. Western blots were performed as described above with the total volume of pulldown and 1:5 (v/v) of the flow-through.

### Proteomics and mass spectrometry analysis

#### Sample preparation

Immuno-isolation of FGF2-positive EVs prior to proteomic analysis was performed by coupling 2 μg FGF2 antibody (AF-233-NA, R&D Systems) to 100 μl Pierce protein A/G magnetic beads (Thermo Fisher Scientific) in PBS/0.001% Tween, kept overnight at 4 °C. Cross-linking was performed with 1 mM final bis(sulfosuccinimidyl)suberate) in 10 mM sodium phosphate, 150 mM NaCl; pH 7.2 for 30 min RT and quenched with 100 mM Glycine. Next, beads were washed three times with PBS/0.001% Tween and incubated with CTL-EVs or FGF2-EVs in PBS overnight at 4 °C (1E + 11p/sample, with a total of 5 replicates representing five different EV preparations from two different DF lines). EV flow-through fractions were collected after spin down and EV pull-down fractions were washed four times with PBS/Tween 0.001%. For EV PD samples, beads were incubated with 100 µl of 8 M urea, 200 mM ammonium bicarbonate (ABC) for 15 min at 1200 rpm and at 37 °C. After spin down, the supernatants were collected and proteins were then reduced with 100 µl of 10 mM dithiothreitol at 57 °C for 30 min and alkylated with 35 µl of 55 mM iodoacetamide in the dark at room temperature for 30 min. Before digestion, samples were diluted with 200 mM ABC to reach a concentration of 1 M urea and 0.4 µg trypsin/Lys-C (Promega) was added twice at 37 °C, for 2 h first and then overnight. EV FT samples (450 µl in PBS) were vacuum concentrated to dryness (using a Savant Centrifuge SpeedVac concentrator) and then suspended in 100 µl of 8 M urea, 200 mM ABC. Proteins were reduced, alkylated and Trypsin/Lys-C digested as previously describe for exosomes samples. Digested samples were loaded into custom-made C18 StageTips packed by stacking one AttractSPE^®^ disk (#SPE-Disks-Bio-C18-100.47.20 Affinisep) and 2 mg beads (#186004521 SepPak C18 Cartridge Waters) into a 200 µl micropipette tip for desalting. Peptides were eluted using a ratio of 40:60 MeCN:H_2_O + 0.1% formic acid and vacuum concentrated to dryness. Peptides were reconstituted in injection buffer (0.3% TFA) before nano-LC–MS/MS analysis.

#### LC–MS/MS analysis

Liquid chromatography using an RSLCnano system (Ultimate 3000, Thermo Scientific) coupled online to an Orbitrap Fusion Tribrid mass spectrometer (Thermo Scientific) was performed. Peptides were trapped on a C18 column (75 μm inner diameter × 2 cm; nanoViper Acclaim PepMapTM 100, Thermo Scientific) with buffer A (2/98 MeCN/H_2_O in 0.1% formic acid) at a flow rate of 4.0 µl/min over 4 min. Separation was performed on a 50 cm × 75 μm C18 column (nanoViper Acclaim PepMapTM RSLC, 2 μm, 100 Å, Thermo Scientific) regulated to a temperature of 55 °C with a linear gradient of 5–26% buffer B (100% MeCN in 0.1% formic acid) at a flow rate of 300 nl/min over 160 min for the exosome flow through and with a linear gradient of 5–25% buffer B over 100 min for the IPs exosome. Peptides were ionized by a nanospray ionization ion source at 2.2 kV. Full-scan MS in the Orbitrap was set at a scan range of 400–1500 with a resolution at 120,000. Ions from each full scan were fragmented in higher energy collisional dissociation mode and analyzed in the linear ion trap in rapid mode. For screening we selected ions with a charge state from 2+ to 7+. Normalized collision energy was set to 30, AGC target to 20,000 and the dynamic exclusion of 30 s.

#### Data processing protocol

For identification, the data were searched against the Homo Sapiens (UP000005640) UniProt database and a databank of the common contaminants using Sequest HT through proteome discoverer (version 2.2). Enzyme specificity was set to trypsin and a maximum of two miss cleavage sites were allowed. Oxidized methionine, carbamidomethylation of cysteins and N-terminal acetylation were set as variable modifications. Maximum allowed mass deviation was set to 10 ppm for monoisotopic precursor ions and 0.6 Da for MS/MS peaks. The resulting files were further processed using myProMS v3.9.2^67^, https://github.com/bioinfo-pf-curie/myproms). FDR calculation used Percolator^68^ and was set to 1% at the peptide level for the whole study. For qualitative protein analysis, proteins were identified from a least of three distinct peptides in best analysis in five replicates. Label free quantification was performed using peptide extracted ion chromatograms (XICs) computed with MassChroQ version 2.2.1^69^. For protein quantification, XICs from proteotypic peptides shared between compared conditions (TopN matching) were used; missed cleavages and peptides modifications were not allowed. Median and scale normalization was applied on the total signal to correct the XICs for each biological replicate (N = 5). To estimate the significance of the change in protein abundance, a linear model (adjusted for peptides and biological replicates) was performed and p-values were adjusted with a Benjamini–Hochberg FDR procedure. Proteins with at least three total peptides in all replicates (n = 5), were displayed in volcano plots. Unique proteins with at least three total peptides in all replicates were considered. Proteins with a fold > 1 enrichment and an adjusted p-value ≤ 0.05 in at least one comparison were considered significantly enriched in the PD-FGF2-EVs fraction. Of these 824 proteins, 391 proteins were found to correlate in both comparisons (parameters: fold change ≥ 1, all peptides ≥ 3 and p-value ≤ 0.05 in at least one quantification). Proteins selected using these criteria were further analyzed with Excel (Suppl. File 1). The mass spectrometry proteomics data have been deposited to the ProteomeXchange Consortium (http://proteomecentral.proteomexchange.org) via the PRIDE partner repository^70^ with the dataset identifier PXD033141.

### Proliferation assay

DFs were seeded in 96-well plates at a density of 1,000 cells/well. Two days later, EVs were added to the wells (8E + 8p in 100 μl per well) or FGF2 (1–100 ng/ml). For stability experiments FGF2 (1 ng per 100 μl) were incubated with EVs (8E + 8p) for 1 h on ice before incubation at 37 °C for 24 h before cell stimulation. On day 3, 5-Bromo-2′-deoxy-uridine (BrdU) was added to the wells for 16 h incubation. BrdU labeling was then conducted using the cell proliferation ELISA BrdU Labeling and Detection Kit III kit (Roche, Merck), according to the manufacturer’s instructions. Cells were fixed and incubated with the primary antibody anti-BrdU for 30 min at 37 °C. Prior to using the ABTS substrate, a secondary antibody coupled with a peroxidase was added for 30 min at 37 °C. Absorbance was then recorded at 405 nm.

### RNA-seq

RNA was extracted using the RNEasy Mini kit with gDNA eliminators columns (QIAGEN). An RNA-Seq experiment was performed with 500 ng total RNA as starting material (n = 2 for each cell type), to obtain double-stranded cDNA as previously described^71^. After purification with the MinElute Reaction Cleanup Kit (28206, QIAGEN), 3 ng of ds-cDNA were processed for library construction using the KAPA Hyper Prep Kit (KK8504, KAPA Biosystems, Roche) according to the standard protocol except that a 15 min USER enzyme (M5505L, NEB, Ipswich, MA, USA) incubation step was added before library amplification. The prepared libraries were quantified with the KAPA Library Quantification Kit (KK4844, KAPA Biosystems, Roche), and then sequenced in a paired-ended manner using a NextSeq 500 (Illumina) according to standard Illumina protocols. Approximately 15–25 × 10^6^ reads were sequenced for each sample. Sequencing reads were aligned to human genome assembly hg19 (NCBI version 37) using STAR 2.5.0 with default options. Genes with the mean of DESeq2-normalized counts (“baseMean”) > 10 were considered to be expressed^72^. Differential gene expression was defined as adjusted P value (padj) < 0.05, and fold change > 2. Functional gene ontology annotation of genes was performed with DAVID^73^.

### Real-time PCR

RNA was extracted using the RNEasy Mini kit (QIAGEN) and cDNA were synthetized from 1 μg of RNA using the iScript cDNA synthesis kit (Bio-Rad). Quantitative PCR were performed in triplicate using the 2 × SYBR Green PCR Master Mix (Absource Biotools). Expression of each gene was calculated using the 2−ΔΔCt method. Results are presented as fold change normalized to GAPDH house-keeping gene and relative to control. Primers used were: hTHBD-FW (ACATCCTGGACGACGGTTTC), hTHBD-Rev (AGATGCCTATGAGCAAGCCC), hESM1-FW (ACCTTCGGGATGGATTGCAG), hESM1-Rev (GATGCCATGTCATGCTCCGT), hMMP1-FW (TCCCAGCGACTCTAGAAACA), hMMP1-Rev (TTCAATCCTGTAGGTCAGATGTGT), hPODXL-FW (TCCCAGAATGCAACCCAGAC), hPODXL-Rev (AGCCACGGTAGTGTTGACTG).

### In vitro wound healing

Eight thousand DF cells were seeded on each side of an IBIDI culture insert (IBIDI, GmbH) in 24-well plates. Four–five days later, upon confluence, the insert was removed and following washing, 1 ml of DMEM with 10% EV-depleted FCS was added. EVs (1E + 9p) were added to the wells and images of the initial gap were taken. Forty-eight hours later, the cells were fixed and stained with hematoxylin/eosin. Closure of the gap, defined as the percent of the initial gap that was covered with cells at 48 h, was analyzed using the ImagePro software.

### In vivo wound healing

Young (9–10 weeks-old) C57Bl/6 NRj female mice were anesthetized by isoflurane inhalation, then clipped and shaved. Full thickness wounds were performed bilaterally using a dermal biopsy 5 mm punch as described in Rhea and Dunnwald^74^. Wounds were located on the lower part of the back of mice and left without dressings to heal. Following wound (D0), 1.5E + 9 particles (or PBS or 500 ng FGF2) in a volume of 125 μl were given per mice, of which 25 μl were topically applied on the wound and 100 μl were injected in four locations surrounding the wound. Pictures were taken daily from day (D) 0 to D5 as well as on D7 and used for wound area quantification. Mice were sacrificed at D7.

### SiRNA transfection

DF cells were transfected with a mix of 25 nM siFGFR1, siFGFR2 and siFGFR3 (smartpool of four sequences, OnTarget Plus, Dharmacon) or siCTRL (scrambled sequence). Three days later, cells were seeded in 96-well plates (1.5 × 10^3^ cells/well), and on the fourth day were treated with FGF2-EVs (8.10E + 8p/well). The following day BrdU was added for 18 h incubation, followed by labeling using the BrDU Labeling and Detection Kit as described above in the proliferation assay section. To confirm FGFR knockdown, cells were collected on days 1 and 3 and western blot for FGFR1, FGFR2 and FGFR3 was performed as described above.

### Ethical statement

The experiments with human samples were performed following receipt of informed consent from the patients, in accordance with the Helsinki Declaration and approved by the INSERM ethical committee. The experiments with mice were approved by the INSERM ethical committee and followed the recommendations of the ARRIVE guidelines. All experiments in the study were conducted according to the relevant guidelines and regulations.

## Supplementary Information

Supplementary Information 1.

Supplementary Information 2.

Supplementary Information 3.

## Data Availability

The data that support the findings of this study are available upon request. The mass spectrometry proteomics data have been deposited into the ProteomeXchange Consortium (http://proteomecentral.proteomexchange.org) via the PRIDE partner repository^70^ with the dataset identifier PXD033141. The RNAseq data have been deposited to GEO database (GEO accession GSE212873: go to https://www.ncbi.nlm.nih.gov/geo/query/acc.cgi?acc=GSE212873)
