# Supplementary material for: Fibroblast growth factor-2 bound to specific dermal fibroblast-derived extracellular vesicles is protected from degradation
Source: Sci Rep. 2022 Dec 22;12:22131. doi: 10.1038/s41598-022-26217-8 (PMC9780220; doi:10.1038/s41598-022-26217-8)

**Fibroblast Growth Factor-2 bound to specific dermal fibroblast-derived extracellular vesicles is protected from degradation**

Isabelle Petit, Ayelet Levy, Soline Estrach, Chloé C. Féral, Andrea Gonçalves Trentin, Florent Dingli, Damarys Loew, Jieqiong Qu, Huiqing Zhou, Clotilde Théry, Céline Prunier, Daniel Aberdam and Olivier Ferrigno

**Supplementary information**

**Suppl. Figure 1: Characterization of the DF-EVs**

**Suppl. Figure 2: Proteome analysis of CTL- and FGF2-EVs by mass spectrometry**

**Suppl. Figure 3: FGF2 binding to EVs from different cell lines**

**Suppl. Figure 4: Activated genes by FGF2-EVs in DFs**

**Suppl. Figure 5: Calibration experiments**

**Original immunoblots**

**Additional files:**

**Suppl. File 1: List of specific proteins in FGF2-EVs.** A total of 824 proteins were significantly more expressed-or unique- in the PD-FGF2-EVs fraction either compared to FT-FGF2-EVs or compared to PD-CTL-EVs. Among these 824 proteins, 391 proteins were found upregulated in both comparisons. Proteins highlighted in green or yellow display a p-value > 0.05. Proteins known to bind FGF2 are highlighted in red.

**Suppl. File 2: List of FGF2-EV- activated genes identified by RNA-seq.**


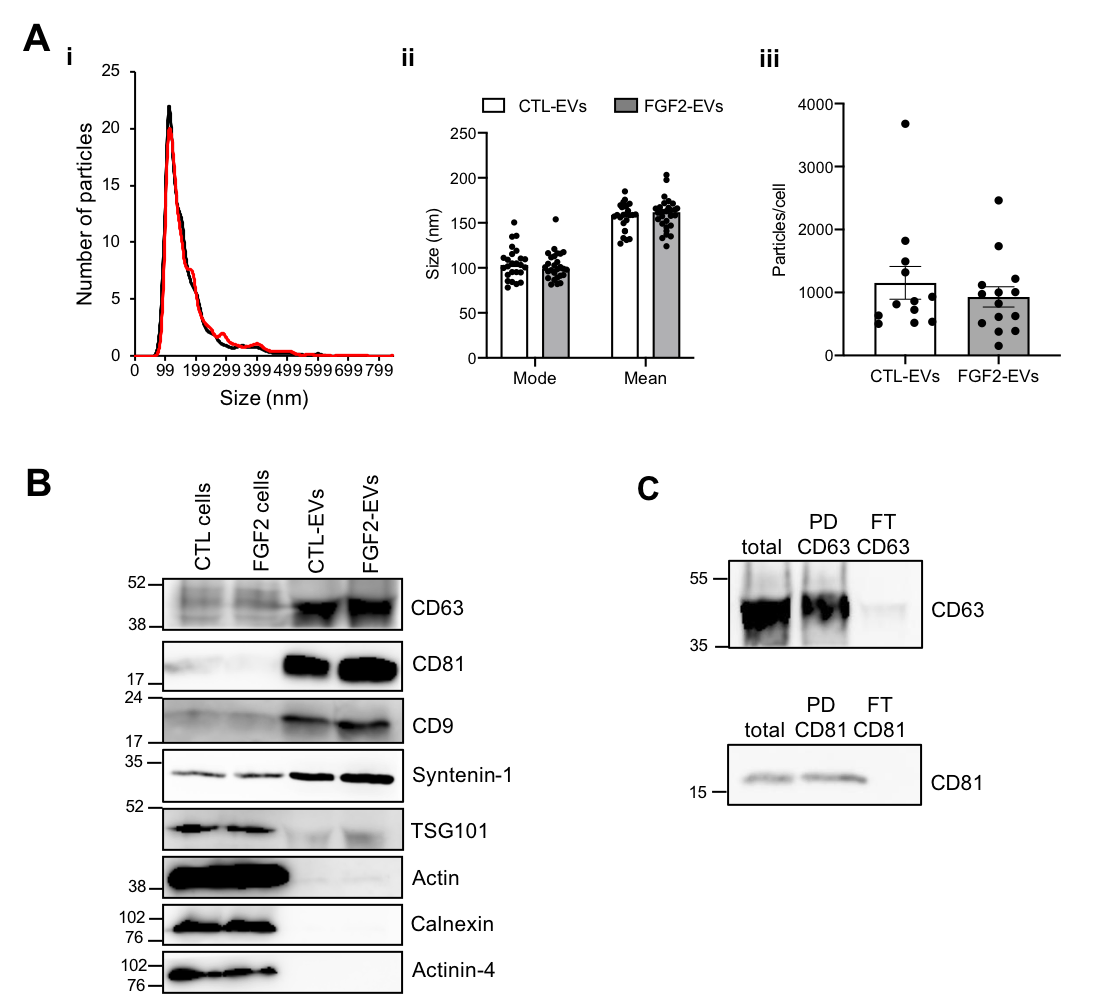


**Suppl. Figure 1:** **Characterization of the DF-EVs**

(**A**) EVs were isolated from dermal fibroblasts treated/not treated with FGF2 (100 ng/ml) and analyzed by Nanoparticle Tracking Analysis. Similar profiles of particle distribution (i, black line=CTL-EVs, red line =FGF2-EVs), median size (ii) and number of particles secreted per cell (iii) were observed for CTL-EVs and FGF2-EVs (p = 0.18, student’s *t-*test, *n*=8). (**B**) Western blot performed with 30 μg proteins for selected proteins revealed that both CTL-and FGF2-EVs contain proteins that are commonly found in EVs (CD63, CD81, CD9, syntenin-1 and TSG101). Both CTL-EVs and FGF2-EVs lacked endoplasmic reticulum calnexin and cytoplasmic actinin-4, which are known to be absent in exosomes and small EVs, but can be present in larger EVs. (**C**) Validation of EV immune-isolation with beads coupled with anti-CD63 or anti-CD81 antibody. Pull-down (PD) and flow-through (FT) fractions were analyzed. Blots are from one representative experiment. Original blots are shown in suppl. Information.


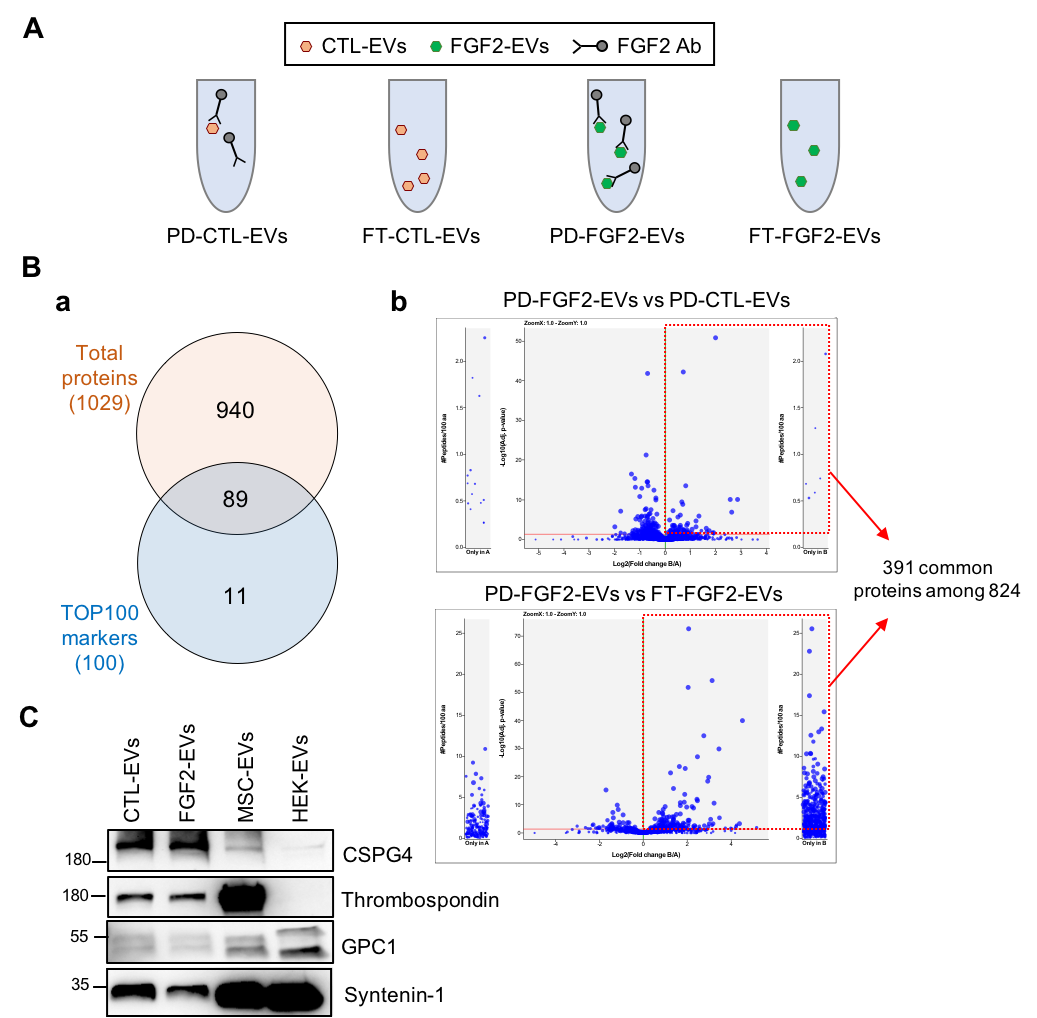


**Suppl. Figure 2:** **Proteome analysis of CTL- and FGF2-EVs by mass spectrometry**

(**A**) Schema of immuno-isolation performed on CTL-EVs and FGF2-EVs using beads coupled to FGF2 antibody. Four fractions were obtained: pull-down (PD) and flow through (FT) for CTL-EVs and FGF2-EVs and named PD-CTL-EVs, FT-CTL-EVs, PD-FGF2-EVs and FT-FGF2-EVs. (**B**) Samples were processed for mass spectrometry analysis and data processing. (**a**) Venn analysis of Top100 EV markers described in Exocarta compared with the total proteins identified from at least three distinct peptides in the best analysis (1029 proteins). (**b**) Quantitative analysis comparing PD-FGF2-EVs with PD-CTL-EVs and PD-FGF2-EVs with FT-FGF2-EVs. Volcano plots show comparative analysis with the following parameters: proteins quantified with at least three peptides in all replicates. A total of 1508 proteins for PD-FGF2-EVs versus FT-FGF2-EVs and 1475 proteins for PD-FGF2-EVs versus PD-CTL-EVs are displayed. 824 proteins were significantly more expressed or unique in the PD-FGF2-EVs fraction either compared to FT-FGF2-EVs or compared to PD-CTL-EVs (parameters: fold change ≥ 1, and all peptides ≥ 3 and p-value ≤ 0.05 in at least one quantification) (**Suppl. file 1**). Among these 824 proteins, 391 proteins were found to be correlated in both comparisons (parameters: fold change ≥ 1, all peptides ≥ 3 and p-value ≤ 0.05 in at least one quantification) (**Suppl. file 1**). (**C**) Expression of FGF2-binding proteins in CTL-EVs, FGF2-EVs, MSC-EVs and HEK-EVs detected by western blot. Representative blots are shown. Original blots are shown in suppl. Information.


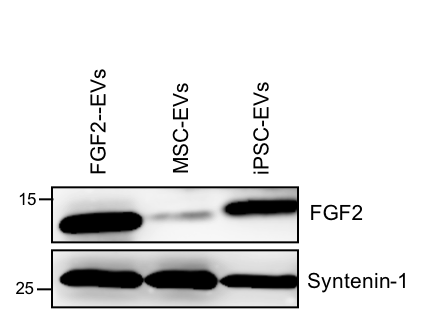


**Suppl. Figure 3:** **FGF2 binding to EVs from different cell lines**

Secreted EVs from DF cells, MSC cells and iPSC cells cultured in 10 ng/ml FGF2 (MSCs) or 100 ng/ml (DFs and iPSCs) were purified and FGF2 expression was examined by western blot (3E+10 p). Blots are representative experiment. Original blots are shown in suppl. Information.


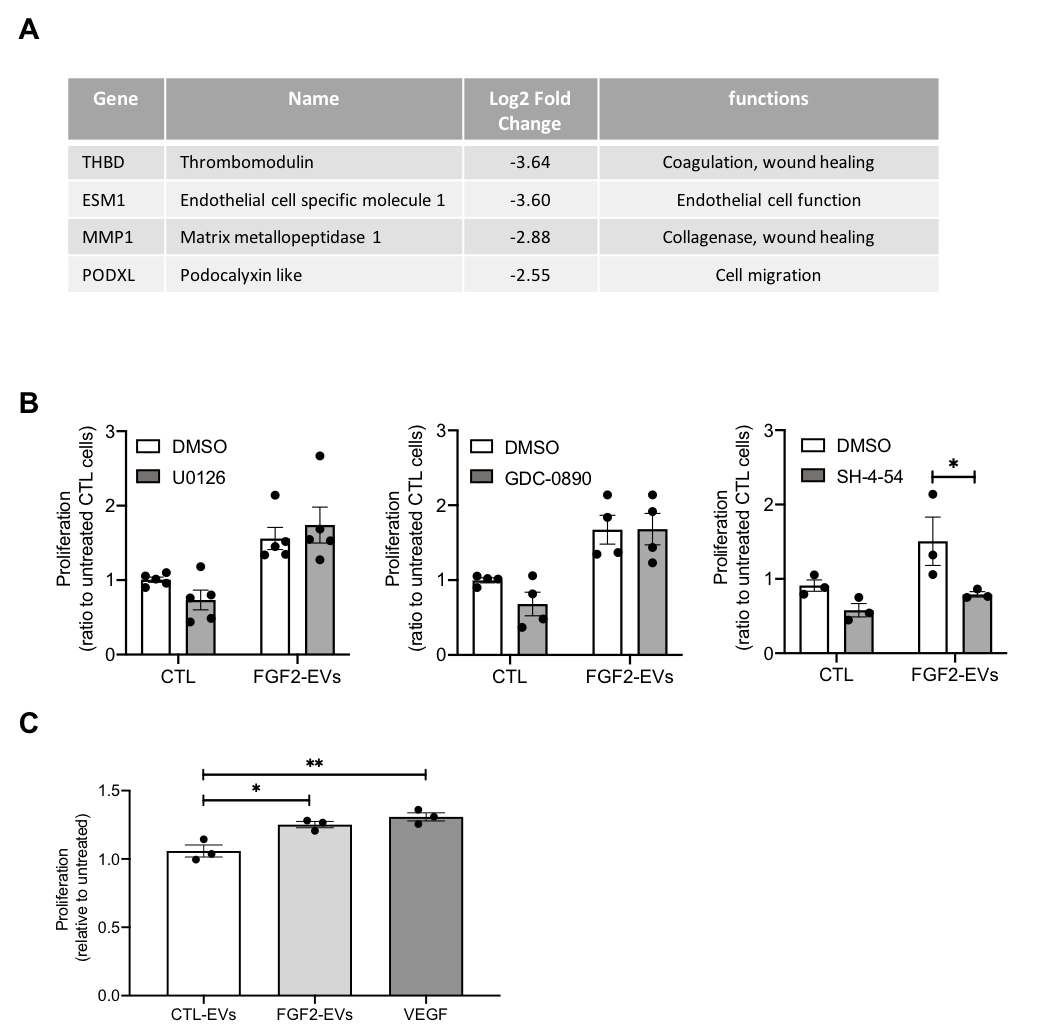


**Suppl. Figure 4:** **Activated genes by FGF2-EVs in DFs**

(**A**) List of the most upregulated genes by FGF2-EVs compared to CTL-EVs. (**B**) The effect of specific inhibitors for the PI3K/AKT pathway (U0126), ERK pathway (GDC-0890) and STAT pathway (SH4-54) was assayed in DF proliferation. Results represent mean of four independent experiments +/- SD. No significant effect was observed for U0126 and GDC-0890. *p value ≤ 0.05, compared using two-way ANOVA (n=4). (**C**) Effect of FGF2-EVs on HDMEC cell proliferation. VEGF serves as positive control. Data are mean of three independent experiments +/- SD. Statistical significance was determined by one-way ANOVA, *<0.05, **<0.01.


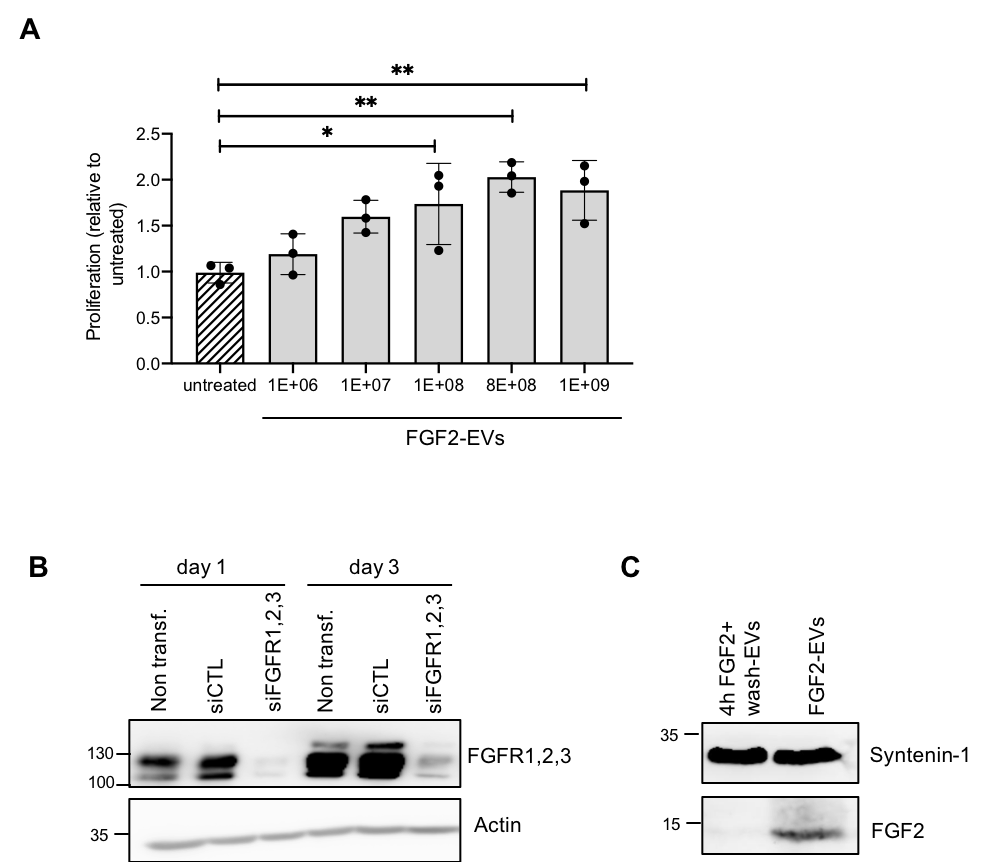


# Suppl. Figure 5: Calibration experiments

(**A**) Dose-response graph for the effect of FGF2-EVs on proliferation. DFs were incubated with increasing amounts of FGF2-EVs in 96 wells and proliferation was measured with BrDU incorporation. Data are mean of three replicates +/- SD. Statistical significance was determined by one-way ANOVA, *<0.05, **<0.01. (**B**) FGFR knockdown by siRNA. DFs were transfected with a mix of 25 nM siFGFR1, siFGFR2 and siFGFR3 or siCTRL (scrambled sequence). Cells were collected on days 1 and 3 and western blot for FGFR1, FGFR2 and FGFR3 was performed to confirm FGFR knockdown. (**C**) Effect of a short FGF2 stimulation on FGF2 localization on EVs. DFs were stimulated for 4h with FGF2 (100 ng/ml), washed with PBS and incubated with medium for 44h. EVs were isolated and FGF2 expression was examined by western blot. Original blots are shown in suppl. Information.

# Original immunoblots

# Figure 1


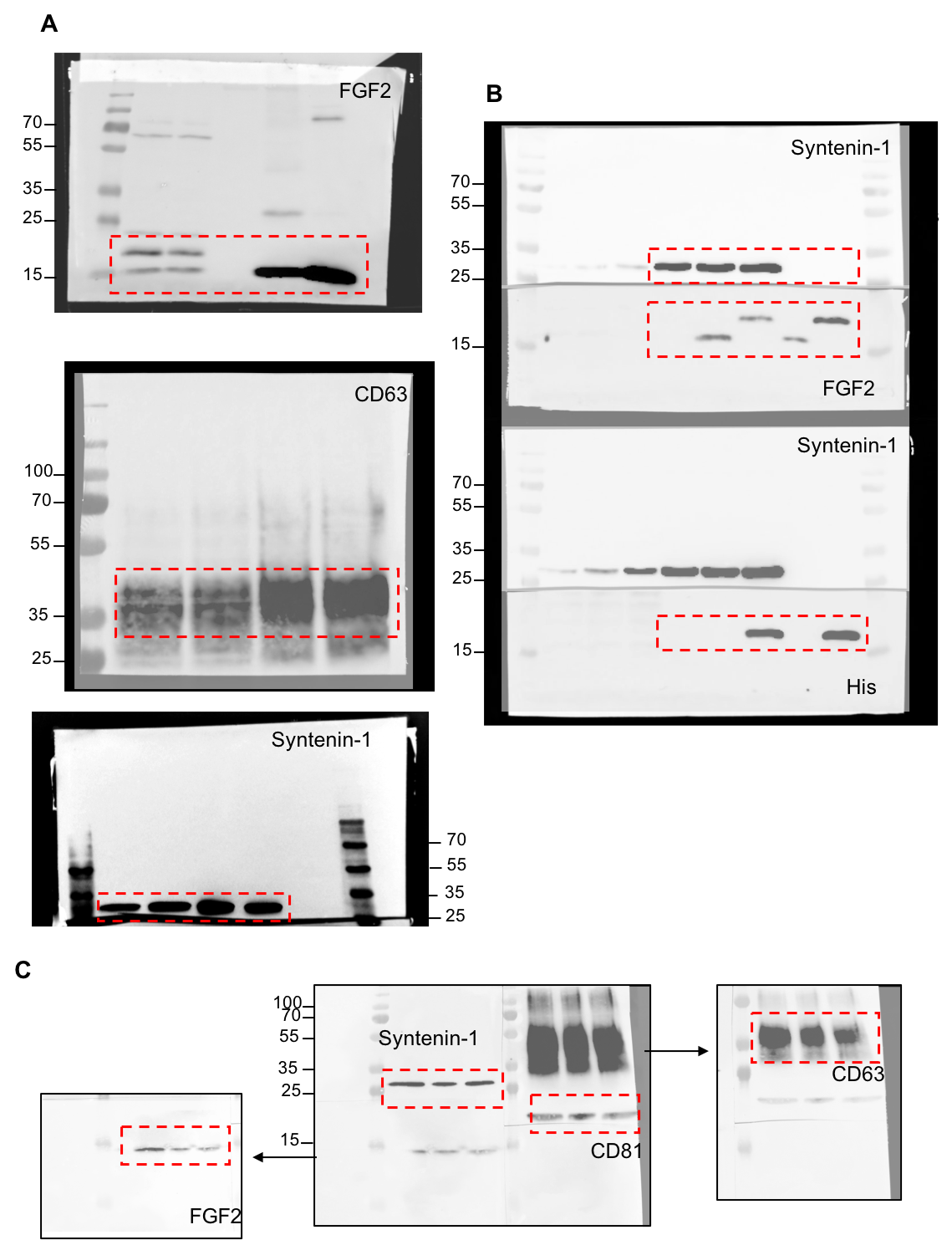


**Figure 2**


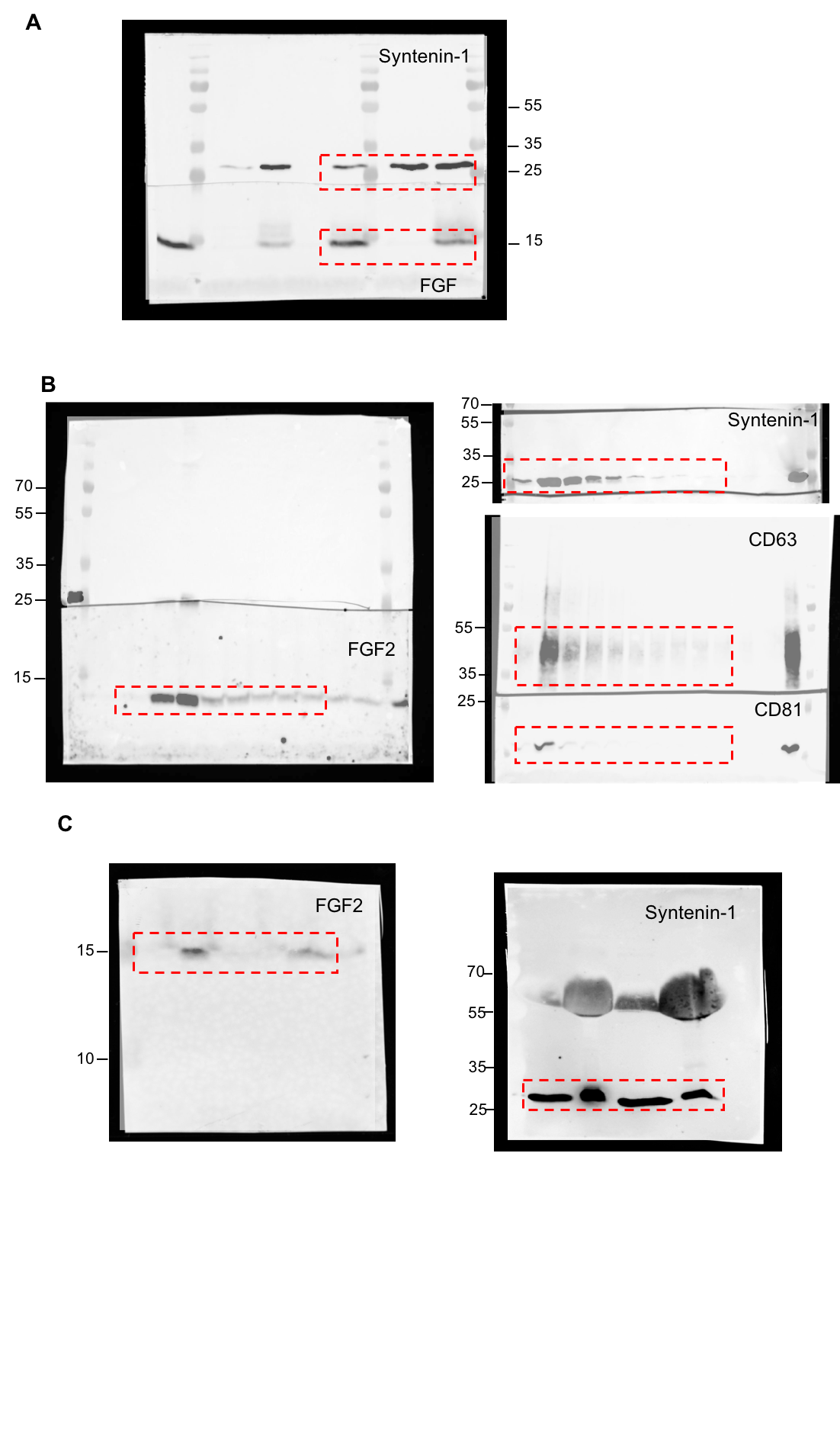


**Figure 3**


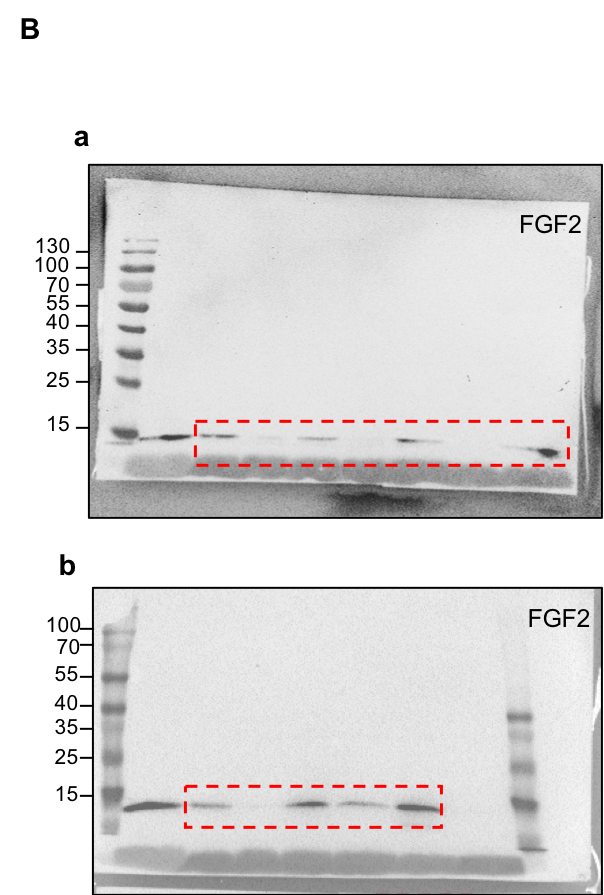


**Figure 4**


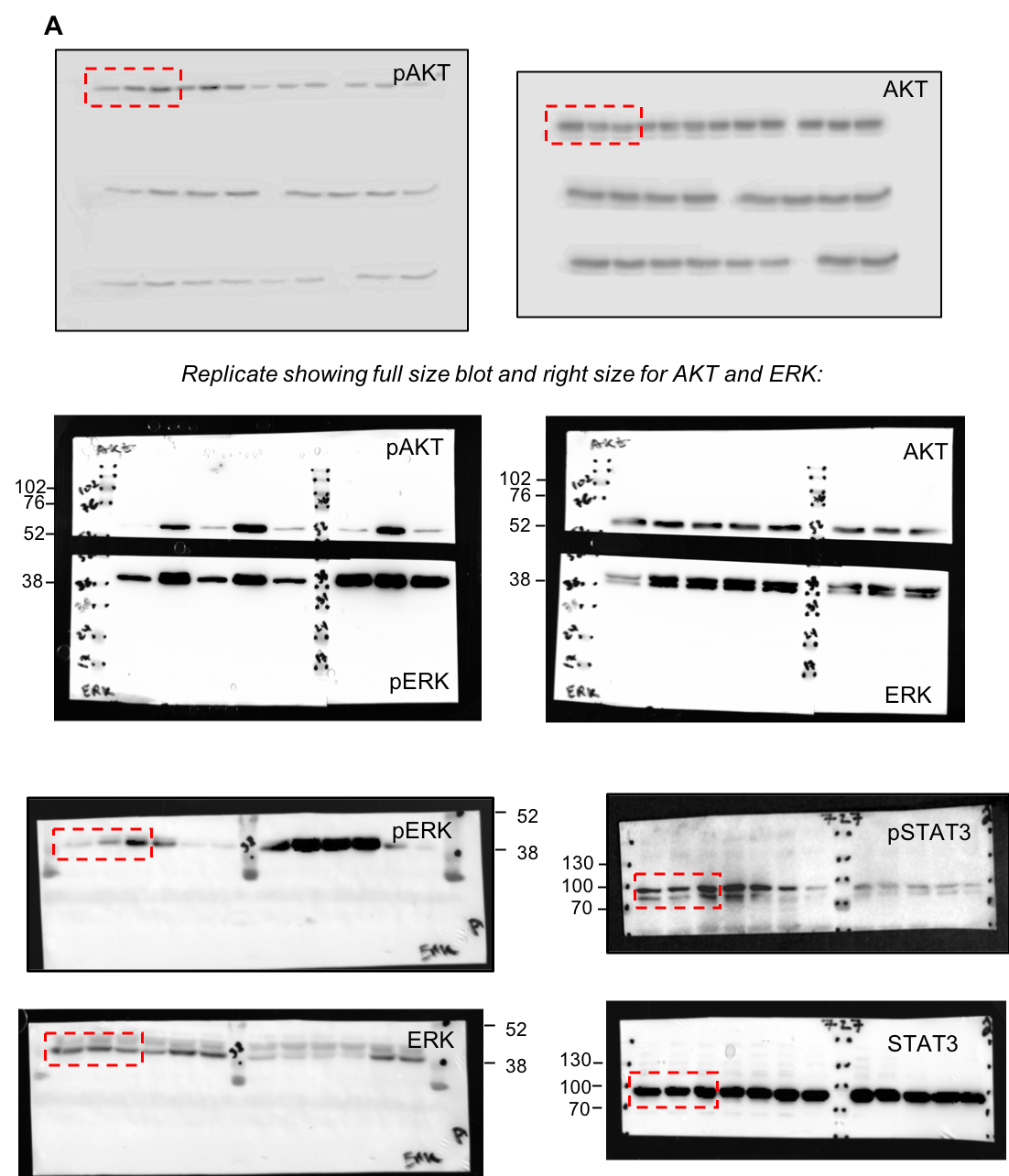


**Suppl. Figure 1**


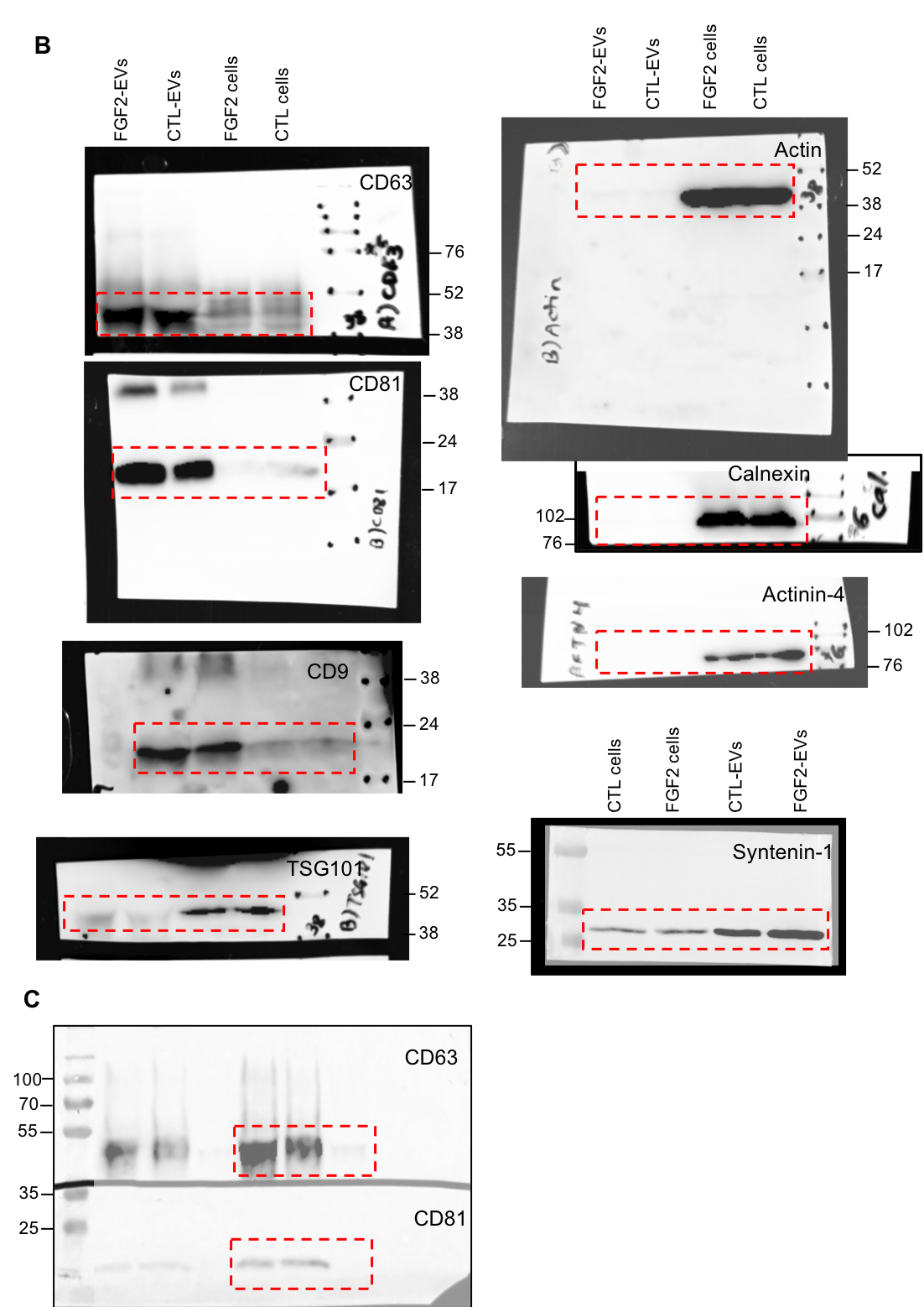


**Suppl. Figure 2**


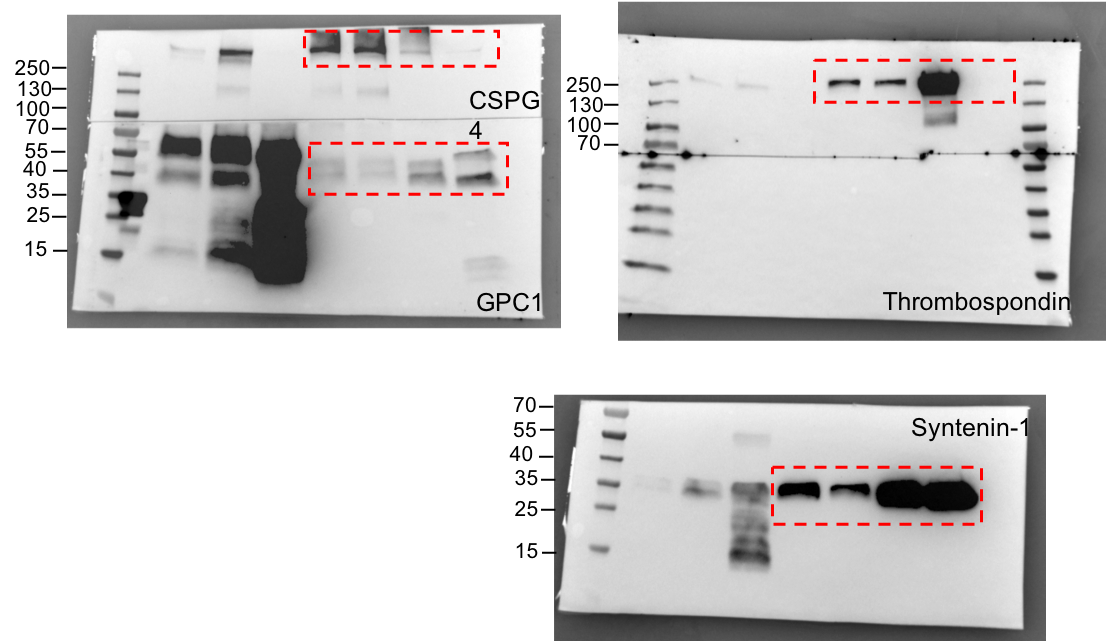


**Suppl. Figure 3**


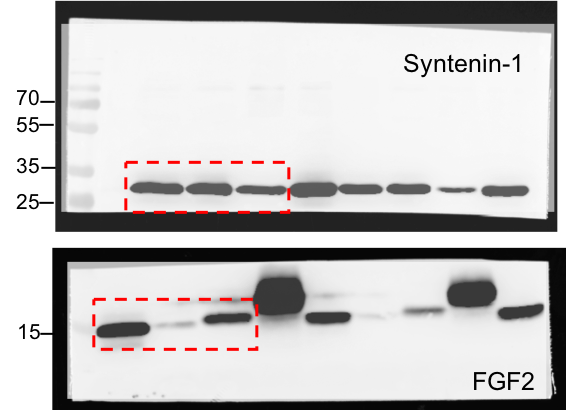


**Suppl. Figure 5**


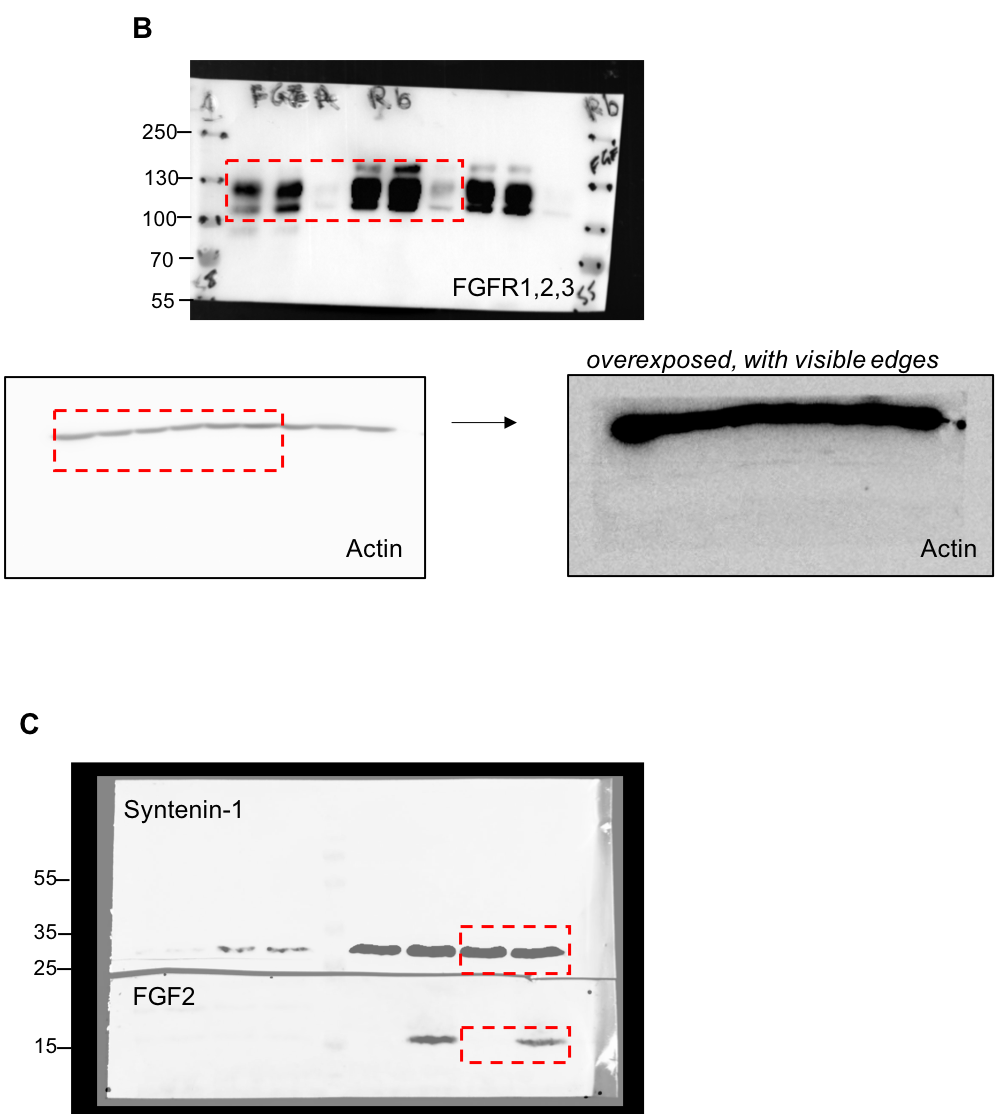

Supplement: Supplementary file 3 — Supplementary Information 3. [file 41598_2022_26217_MOESM3_ESM.docx]
